# Incomplete recovery of tree community composition and rare species after 120 years of tropical forest succession in Panama

**DOI:** 10.1111/btp.13275

**Published:** 2023-10-30

**Authors:** Alexander D. Elsy, Marion Pfeifer, Isabel L. Jones, Saara J. DeWalt, Omar R. Lopez, Daisy H. Dent

**Affiliations:** ^1^ Biological and Environmental Sciences University of Stirling Stirling UK; ^2^ School of Natural and Environmental Sciences, Modelling, Evidence and Policy Group Newcastle University Newcastle upon Tyne UK; ^3^ Department of Biological Sciences Clemson University Clemson South Carolina USA; ^4^ Smithsonian Tropical Research Institute Balboa Panama; ^5^ Instituto de Investigaciones Científicas y Servicios de Alta Tecnología (INDICASAT) Clayton Panama; ^6^ Max Planck Institute for Animal Behavior Konstanz Germany; ^7^ Department of Environmental Systems Science ETH Zürich Zurich Switzerland

**Keywords:** alternate successional pathways, chronosequence, forest structure, *Gustavia superba*, rarity, species diversity

## Abstract

Determining how fully tropical forests regenerating on abandoned land recover characteristics of old‐growth forests is increasingly important for understanding their role in conserving rare species and maintaining ecosystem services. Despite this, our understanding of forest structure and community composition recovery throughout succession is incomplete, as many tropical chronosequences do not extend beyond the first 50 years of succession. Here, we examined trajectories of forest recovery across eight 1‐hectare plots in middle and later stages of forest succession (40–120 years) and five 1‐hectare old‐growth plots, in the Barro Colorado Nature Monument (BCNM), Panama. We first verified that forest age had a greater effect than edaphic or topographic variation on forest structure, diversity and composition and then corroborated results from smaller plots censused 20 years previously. Tree species diversity (but not species richness) and forest structure had fully recovered to old‐growth levels by 40 and 90 years, respectively. However, rare species were missing, and old‐growth specialists were in low abundance, in the mid‐ and late secondary forest plots, leading to incomplete recovery of species composition even by 120 years into succession. We also found evidence that dominance early in succession by a long‐lived pioneer led to altered forest structure and delayed recovery of species diversity and composition well past a century after land abandonment. Our results illustrate the critical importance of old‐growth and old secondary forests for biodiversity conservation, given that recovery of community composition may take several centuries, particularly when a long‐lived pioneer dominates in early succession.

Abstract in Spanish is available with online material.

## Graphical abstract

The late stages of tropical forest succession are understudied. Here we analyze tree community recovery over one of the oldest chronosequences in the Neotropics and find recovery of species diversity and forest structure, within 40 and 90 years respectively, but species community compositional recovery is slow, with one plot converging on old‐growth after 120‐years of succession. We also see evidence for an alternate successional pathway, characterized by long‐lived pioneer dominance and dissimilarity to old‐growth communities.

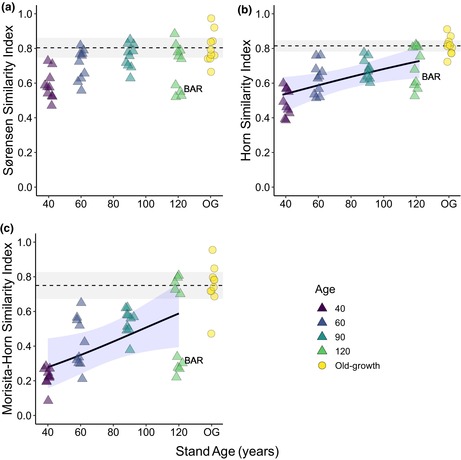

## RESUMEN

Determinar en que medida los bosques tropicales que se regeneran en tierras abandonadas recuperan las características de los bosques primarios es cada vez más importante para comprender su papel en la conservación de especies raras y el mantenimiento de los servicios ecosistémicos. A pesar de ello, nuestro entendimiento sobre la recuperación de la estructura del bosque y la composición de la comunidad a lo largo de la sucesión es incompleta, ya que muchas cronosecuencias tropicales no van más allá de los primeros 50 años de sucesión. En este estudio, investigamos las trayectorias de recuperación del bosque en ocho parcelas de 1 hectárea en estadíos medios y tardíos de la sucesión forestal (40–120 años) y cinco parcelas de 1 hectárea de bosque primario, en el Monumento Natural Barro Colorado (MNBC), Panamá. En primer lugar, verificamos que la edad del bosque tenía un mayor efecto que la variación edáfica o topográfica en la estructura, diversidad y composición del bosque y luego corroboramos los resultados de parcelas más pequeñas estudiadas 20 años antes. La diversidad de especies arbóreas, pero no la riqueza de especies, y la estructura forestal se habían recuperado completamente hasta alcanzar los niveles de bosque primario a los 40 y 90 años, respectivamente. Sin embargo, los bosques secundarios carecían de especies raras y presentaban una escasa abundancia de especies especialistas del bosque antiguo, lo que condujo a una recuperación incompleta de la composición de especies, incluso a 120 años de sucesión. También encontramos pruebas de que el predominio de un pionero longevo en las primeras etapas de la sucesión provocó una alteración de la estructura forestal y retrasó la recuperación de la diversidad y composición de especies más allá de un siglo después el abandono de las tierras. Nuestros resultados ilustran la importancia crítica de los bosques primarios y secundarios más antiguos para la conservación de la biodiversidad, dado que la recuperación de la composición de la comunidad puede llevar varios siglos, especialmente cuando un pionero longevo domina en la sucesión temprana.

## 1 INTRODUCTION

Understanding successional processes during forest recovery is increasingly important, as over 60% of the world's forests are currently regrowing following disturbance (Pugh et al., 2019). In Neotropical forests, many aspects of forest structure and diversity recover rapidly (within the first 50 years of succession), whilst attributes such as aboveground biomass and community composition recover more slowly (Guariguata & Ostertag, 2001; Poorter et al., 2021). For instance, tree species richness can recover 80% of old‐growth values in just 20 years, with complete recovery often within 50 years (Martin et al., 2013; Poorter et al., 2021; Rozendaal et al., 2019). Stem density (Kennard, 2002; Peña‐Claros, 2003) and light availability (Denslow & Guzman, 2000; Lebrija‐Trejos et al., 2011; Nicotra et al., 1999) recover to values akin to old‐growth forests within the first 40 years. In contrast, aboveground biomass can take up to 120 years to recover (Poorter et al., 2021). Community composition is typically the last characteristic of secondary forests to recover to old‐growth levels, with estimates of recovery ranging from 120 years (Poorter et al., 2021), to 210 years (Cole et al., 2014), to several centuries (Rozendaal et al., 2019).

Chronosequences, or space‐for‐time substitutions, are used to study secondary succession in tropical forests (Chazdon et al., 2007). Most research in tropical forest chronosequences has focused on young forests (typically 0–40 years old), while old secondary forests (> 80 years old) are relatively poorly represented in the literature, in part due to difficulties in aging older secondary forest and misclassification of old secondary forests as “undisturbed” or “primary” forests (Brown & Lugo, 1990; Milton et al., 1994). In two recent studies of carbon and biodiversity recovery in tropical forests, only four of 56 Neotropical chronosequences (Rozendaal et al., 2019) and just 12 of 204 tropical forest plots (Martin et al., 2013) included in the analyses were ≥ 80 years old. The poor representation of older plots in syntheses of secondary tropical forest recovery biases our understanding towards young forest stands. It also limits our understanding of processes that take place over long time scales, such as community compositional recovery, and may partly explain the variable recovery rates reported for biomass (Martin et al., 2013; Poorter et al., 2016) and community composition (Cole et al., 2014; Poorter et al., 2021; Rozendaal et al., 2019). Therefore, accurately aged old secondary forest plots are critical for quantifying aspects of tropical forests that recover slowly.

Chronosequence studies often do not adequately account for biotic and abiotic differences among plots (Johnson & Miyanishi, 2008), and so it is necessary to consider environmental variables, in addition to forest age, when looking to explain rates of forest recovery. Myriad factors influence the speed and trajectory of community composition recovery throughout succession (Arroyo‐Rodríguez et al., 2017). At landscape scales, old‐growth forest connectivity, landscape structure and matrix composition can all influence floristic recovery (Arroyo‐Rodríguez et al., 2017; Damschen & Brudvig, 2012; Hernández‐Stefanoni et al., 2011; Laurance et al., 2007). At the local scale, factors such as land use history, topography, soil fertility and liana abundance may determine the tree community (Baldeck et al., 2013; Estrada‐Villegas et al., 2019; Visser et al., 2018), alter forest structure (Cushman et al., 2022; Jucker et al., 2018; Tymen et al., 2016), or even cause alternate successional pathways to develop (Arroyo‐Rodríguez et al., 2017; Jakovac et al., 2021). Alternate successional pathways may result from different land use and land use intensities (Jakovac et al., 2021), disturbance regimes (Mesquita et al., 2015), arrested recovery due to liana dominance (Marshall et al., 2020), or source and disperser limitation leading to priority effects (Dent & Estrada‐Villegas, 2021; Weidlich et al., 2021), ultimately leading to taxonomically and structurally distinct tree communities within a landscape (Jakovac et al., 2015; Mesquita et al., 2015; Norden et al., 2011; Tymen et al., 2016).

The spatial scale of forest sampling also influences the interpretation of patterns in secondary forest succession; plot size, shape, and spatial extent all affect species diversity and stem density (Condit et al., 1996; Güler et al., 2016; Schnitzer et al., 2006). For instance, Chazdon et al. (2023) subsampled 1‐ha tropical forest plots and found that plot sizes less than 0.5‐ha may not accurately capture tree species diversity or evenness. Large plots (≥0.5 ha) typically encompass more heterogeneous environments and are more robust to stochastic events (e.g., treefalls; Schnitzer & Carson, 2001) than smaller plots. However, to understand variable successional pathways, plots need to be distributed across the study landscape and must be located in single aged forest stands (Arroyo‐Rodríguez et al., 2017). Thus, many secondary forest studies prioritize the number and distribution of census plots over individual plot area (see sites included in Rozendaal et al. (2019)).

Community compositional recovery in secondary forests is a complex property to capture, as it is important to identify both rare species of potential conservation concern and dominant species that likely have the largest impact on ecosystem function (Cavanaugh et al., 2014; Hubbell, 2013; ter Steege et al., 2013). Moreover, examining the recovery of generalist, specialist and rare species, alongside community composition, can provide insights into why secondary forest function or composition may not converge on that of old‐growth communities (Boukili & Chazdon, 2017; Lasky et al., 2014; Norden et al., 2017). Rare species in particular are likely to recover slowly (e.g., Goosem et al., 2016), given that they often have poor dispersal ability (Gaston, 1994) and exhibit highly clustered distributions (Condit et al., 2000). Therefore, analyses which incorporate incidence‐, abundance‐ and dominance‐based community composition metrics alongside species classification techniques (e.g., Norden et al., 2017) can provide a more detailed understanding of secondary succession.

Here, we re‐examine tree and palm communities across a chronosequence (40–120 years) in the Barro Colorado Nature Monument (BCNM), central Panama, that has previously been used to investigate recovery in forest structure, diversity, and species composition, as well as multisite comparisons (e.g., Chazdon et al., 2016; Poorter et al., 2016, 2021; Rozendaal et al., 2019). Previous analyses have found that secondary forests in the BCNM regained old‐growth species diversity and richness within 20 years, aspects of forest structure recovered within 70 years and aboveground carbon in >100 years (Denslow, 2000; Denslow & Guzman, 2000; Dent et al., 2013; DeWalt et al., 2003; Jones et al., 2019; Mascaro et al., 2012). They also suggest that secondary forests had not converged on old‐growth species composition for trees that attain canopy or midstory stature (Dent et al., 2013). In this study, we include a recensus c. 20 years after the original (Denslow & Guzman, 2000), with larger plots in each forest stand (1‐ha vs. 0.32‐ha) and three additional old‐growth plots, to address three main objectives: (1) Determine how quickly forest structure, species diversity and community composition (cf. rare, generalist and specialist species) recover following land abandonment; (2) Investigate whether the extended timespan and increased spatial scale of sampling within the chronosequence alters our understanding from previous studies (e.g., Dent et al., 2013); (3) Examine the importance of topography and soil nutrients in separately shaping patterns of structure, diversity, and compositional recovery. This study represents one of the oldest chronosequences in the Neotropics (Buzzard et al., 2016; Rozendaal et al., 2019) and is uniquely situated to examine late‐stage secondary successional forest recovery.

## 2 METHODS

### 2.1 Study area

This study uses data from a secondary forest chronosequence (Denslow & Guzman, 2000) and the 50‐ha ForestGEO plot (Condit, 1998; Hubbell et al., 1999) within the BCNM, central Panama. Our 13 study plots were situated on Barro Colorado Island (BCI; 9^o^08’60” N, −79^o^50’60” W) and surrounding peninsulas (Figure S1). The BCNM receives ~2600 mm of rainfall annually with a distinct dry season between January and May (Paton, 2020). Vegetation in the area is classified as tropical moist forest (Holdridge, 1947); for information on the flora, geology and soils of BCI, see Croat (1978) and Baillie et al. (2006).

The BCNM secondary forest chronosequence comprises eight 1‐ha plots across forest stands that were aged (in 2015) approximately 40, 60, 90, and 120 years since land‐abandonment, with two independent plots per stand age (Jones et al., 2019; Figure S1). The chronosequence was established in 1994 with 0.32‐ha plots (two paired 10 × 160 m transects); these were enlarged to 1‐ha (50 × 200 m) in 2011 with plots positioned to maximize overlap with existing transects (Supporting Information). For one 120‐year‐old plot (Pearson; Table S1), only 0.88‐ha was censused due to an incomplete census in 2014. Forest stand ages were determined from aerial photographs, interviews with residents and existing literature and are accurate to within c. 10 years of land abandonment (Denslow & Guzman, 2000). All secondary forest plots were previously used for pasture, swidden or plantation farming and were undisturbed since abandonment (Denslow & Guzman, 2000; Table S1). The plots were a minimum distance of 1.07 km apart (mean ± SD = 4.39 ± 2.32 km). The 50‐ha ForestGEO plot was predominately undisturbed old‐growth forest (Condit, 1998). Five 1‐ha (100 × 100 m) subplots were selected from within the 50‐ha plot as old‐growth forest comparisons. The 1‐ha subplots were located at the corners and centre of the plot (minimum distance between subplots = 0.32 km, mean ± SD = 0.55 ± 0.25 km).

### 2.2 Data collection

#### 2.2.1 Forest census

Secondary forest plots were surveyed between 2011 and 2016 (Elsy et al., 2023), following ForestGEO vegetation census protocols (Condit, 2008). All trees, palms and shrubs ≥5 cm diameter at breast height (DBH) were measured in each plot, with trees identified to species level where possible (98.3% of individuals identified to species). Data for the old‐growth 1‐ha plots were obtained from the 2015 50‐ha plot census (all individuals identified to species‐level; Condit et al., 2019, 2020).

#### 2.2.2 Environmental descriptions

For environmental variables we examined soil nitrogen (N), phosphorus (P) and slope. Soil inventories were obtained from Jones et al. (2019) for secondary forest plots and Wolf et al. (2015) for the old‐growth 50‐ha plot. Both followed ForestGEO soil sampling protocols (ForestGEO, 2010) and soil cores were sampled to 10 cm depth in regularly spaced grids, with 10 cores per secondary forest plot and 12 per old‐growth plot. We averaged soil N and P per plot. For the detailed methodology, see ForestGEO (2010), Jones et al. (2019) and Wolf et al. (2015). Topographic data were obtained by merging a 1 m x 1 m resolution digital elevation model (DEM) covering the BCNM and a 5 m x 5 m resolution DEM covering Central Panama (STRI, 2020a, 2020b). The merged DEM was upscaled to 10 m x 10 m resolution using the “aggregate” function in the “raster” R package (Hijmans, 2022), and mean values of terrain slope per plot were calculated.

### 2.3 Statistical analysis

#### 2.3.1 Forest structure, species richness, and diversity

Forest structural characteristics were computed as stem density and basal area per ha, including unidentified trees and multiple stems per individual tree (total number of stems = 15,281). A correction factor was applied to stem density and basal area values for the Pearson plot (0.88‐ha multiplied by 1.136) to enable between‐plot comparisons at 1‐ha scale. Rarefied species richness (calculated on the minimum number of identified individual trees per plot, *n* = 807), Simpson's diversity index (generated using the “simpson.unb” function to account for differing sample sizes) and Pielou's evenness index were calculated per plot using the “vegan” package (Oksanen et al., 2022) for all identified individual trees (*n* = 12,836). Extrapolated species richness curves were also calculated using the “iNEXT” R package and 100 bootstrapped iterations (Chao, Gotelli, et al., 2014; Hsieh et al., 2016). Richness values were conservatively extrapolated up to double the minimum number of trees per plot (*n* = 1614) and extrapolations were included to utilize all available data and allow robust comparisons between plots (Colwell et al., 2012).

We modeled changes in forest structure and diversity metrics using generalized linear models (GLMs) or beta regressions (Cribari‐Neto & Zeileis, 2010), using the conditional distribution that best fit the data (see Supporting Information and Table S2) and the “DHARMa” package to examine model residuals (Hartig, 2022). We used a two‐step approach, first focusing on modeling secondary forest plots solely against stand age (Tables S2, S3). Old‐growth plots were excluded from these models because they could not be assigned a stand age. Secondly, we modeled all plots (including old‐growth) against mean soil nitrogen, phosphorus and slope and fit full models with all three environmental variables. Model selection was carried out using the “dredge” function (“MuMIn” package; Bartoń, 2022) to select the model with the lowest AICc. Model averaging was conducted on models with ΔAICc ≤4 from the minimum AICc model (Tables S4, S5; Anderson & Burnham, 2004). Differences in forest structure and diversity between old‐growth and secondary forest plots were tested using two‐sample Wilcoxon tests (Table S6).

#### 2.3.2 Species community composition

We examined species community composition recovery by calculating pairwise similarity indices comparisons between secondary forest plots and old‐growth plots, following Norden et al. (2017). We followed the Hill number framework and calculated the incidence‐based Sørensen similarity index (*q* = 0) and the abundance‐based Horn (*q* = 1) and Morisita‐Horn (*q* = 2) similarity indices (Chao, Chiu, et al., 2014; Jost et al., 2011) using the “SimilarityMult” function from “SpadeR” (Chao et al., 2016). Each index allowed us to examine an aspect of community recovery: the Sørensen similarity index is based on species presence‐absence in a community; the Horn similarity index weights species by their relative abundance and the Morisita‐Horn similarity index is heavily influenced by species dominance (Jost et al., 2011; Norden et al., 2017). We modeled pairwise similarity indices comparisons against stand age (fixed effect) using generalized linear mixed models (GLMMs) with a beta distribution (Table S7), and plot as a random effect (random intercept only), through the “glmmTMB” package (Brooks et al., 2017). We modeled plot as a random effect to account for non‐independence of each secondary‐old‐growth pairwise comparison (*n* = 5 per eight secondary forest plots). The community composition GLMMs deviated from the expected values in residual versus predicted plots, likely due to the influence of one 120‐year‐old outlier plot, Barbour. The random effect variance was not reliable, and we do not report conditional *R* ^*2*^ estimates derived from it. We report the overall model, however, given that the trend of community composition recovery is apparent from data visualization (Figure 2).

We used non‐metric multidimensional scaling (NMDS) to investigate community composition, via the “metaMDS” function in “vegan” (Oksanen et al., 2022), for each similarity index with 10,000 random starts. The lowest stress run was used for each index; however, the NMDS based on the Horn similarity index had high stress (0.244), and we therefore only examined the NMDS based on Sørensen and Morisita‐Horn indices (stress <0.20).

#### 2.3.3 Species specialism and rarity

We classified species as secondary forest specialists, old‐growth specialists, generalists or too rare to classify, based on the multinomial model developed by Chazdon et al. (2011). This model estimates true relative species abundances in each forest type, assuming random sampling error, and takes into account potentially undetected species to calculate relative species abundances, which are compared to determine the species classification (Chazdon et al., 2011). We followed recommendations in Chazdon et al. (2011) and used a 2/3 specialization threshold, and *p* = .00125, to conservatively classify shared species as habitat specialists (secondary forest or old‐growth) or generalists through the “clamtest” function in “vegan” (Oksanen et al., 2022). Those species identified as “too rare to classify” were those found at too low abundance to assign to another classification, we term these species “rare” species hereafter. We tested if the number of rare species differed between forest type using a Wilcoxon rank‐sum test. We confirmed that differing sample areas (old‐growth = 5‐ha, secondary forest = 8‐ha) had no effect on the results by re‐running the multinomial model on all combinations of five secondary plots (*n* = 6720) against the five old‐growth plots (Figure S2).

All analyses were carried out in R (version 4.2.2; R Core Team, 2022) with data cleaning completed using the “tidyverse” (Wickham et al., 2019). Model predictions were generated using the “effects” package (Fox & Weisberg, 2019).

## 3 RESULTS

### 3.1 Forest structure, species richness and diversity

**FIGURE 1:**
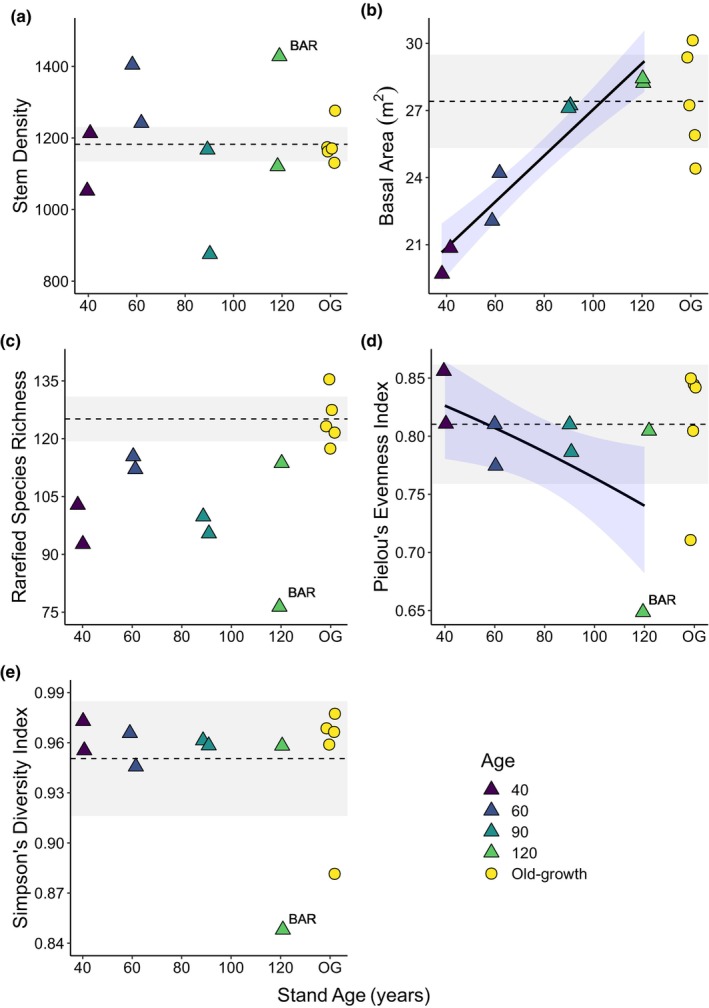
Forest structure, richness and diversity metrics plotted against stand age. (a). Stem density; (b). Basal area; (c). Rarefied species richness; (d). Pielou's evenness index and (e). Simpson's diversity index. Mean values of old‐growth plots are indicated by the dashed lines; gray shading indicates 95% confidence intervals. Model predictions (±95% C.I.) are plotted in blue shading for models with significant age effects. The 120‐year‐old plot “Barbour” is labeled with “BAR” in each plot because it is a frequent outlier.

A total of 277 species and 12,836 individual trees, palms and shrubs ≥5 cm DBH were surveyed across eight secondary and five old‐growth 1‐ha plots (Table S1). We found no evidence of mean N, P or slope predicting structural or diversity metrics across secondary and old‐growth plots (Table S5), and therefore focus only on effects of forest age. Stem density was more varied among secondary than old‐growth forest plots (Figure 1a), but there was no effect of stand age (*p* = .886; Table S3). Basal area significantly increased with stand age (*p* < .001, *R* ^*2*^ = 0.911) and recovered to the mean old‐growth level by mid‐to‐late stages of succession (~90 years, Figure 1b). Rarefied species richness was significantly lower in secondary forest plots compared to old‐growth forest (Wilcoxon test: *p* = .002), but no effect of stand age on richness was found (Table S3; Figure 1c). Extrapolated species richness values were also significantly lower in secondary forest plots (Wilcoxon test: *p* = .011), although both 60‐year‐old plots, and one 120‐year‐old plot, were approaching old‐growth values (Figure S3). Simpson's diversity index had recovered to old‐growth levels by the start of the chronosequence (Figure 1e). Species evenness had also recovered to old‐growth levels in the youngest plots (40 years) in the chronosequence, and then declined significantly with stand age (*p* = .028, Figure 1d). One 120‐year‐old plot, Barbour, heavily influenced the decline in evenness over stand age (Figure 1d). Barbour was dominated by *Gustavia superba* (Lecythidaceae), which comprised 35.7% of all trees, palms and shrubs ≥5 cm in the plot (Table S8). This dominance was higher than in all other secondary forest plots (mean dominance = 12.3%), and only one old‐growth plot had a similar rank‐abundance relationship (Figure S4), due to high dominance by the understory tree *Faramea occidentalis* (Rubiaceae) (33.0%; Table S8).

### 3.2 Species community composition

**FIGURE 2:**
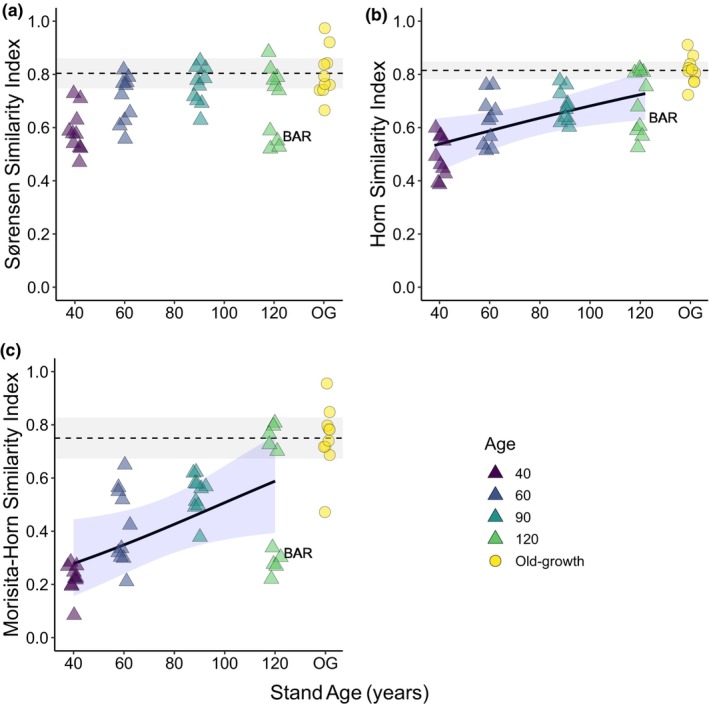
Pairwise similarity index comparisons between each secondary forest plot and old‐growth plots plotted against stand age for: (a). Sørensen similarity index, (b). Horn similarity index, and (c). Morisita‐Horn similarity index. The black line and blue 95% confidence intervals indicate the GLMM predictions for pairwise secondary‐old‐growth comparisons. Old‐growth pairwise comparisons are graphed for comparison but are not included in the model. Mean values of pairwise old‐growth similarity comparisons are indicated by the dashed lines; gray shading indicates 95% confidence intervals. The 120‐year‐old plot “Barbour” is labeled with “BAR” in each plot because it is a frequent outlier.

The community composition of secondary forests showed evidence of convergence on old‐growth composition across all pairwise similarity indices (Figure 2). Old‐growth plots were ~80% similar to each other in species composition regardless of similarity index. Pairwise incidence‐based Sørensen similarity comparisons converged fastest, with recovery within 60 years of forest regrowth, and there was no significant effect of stand age throughout the chronosequence (*p* = .144; Table S7). In contrast, abundance‐based Horn and Morisita‐Horn similarity comparisons significantly increased with stand age (*p* = .013, .029) and only one 120‐year‐old plot had converged on mean old‐growth similarity (Figure 2b, c). This indicates that species typical of old‐growth forests are present early in succession, but it takes time for the relative abundance of those species to attain patterns seen in old‐growth forests. For all indices, one 120‐year‐old plot, Barbour, was less similar to old‐growth than expected from the community composition trajectories. The NMDS ordinations showed these differences between incidence and abundance‐based indices, with plots in the Sørensen NMDS grouped more closely together in ordination space than the Morisita‐Horn NMDS (Figure S5). Both NMDS plots showed secondary plots became closer to old‐growth plots in ordination space with increasing age, demonstrating increasing similarity to old‐growth. However, none of the secondary forest plots were within the old‐growth cluster (Figure S5).

### 3.3 Species specialism and rarity

**FIGURE 3:**
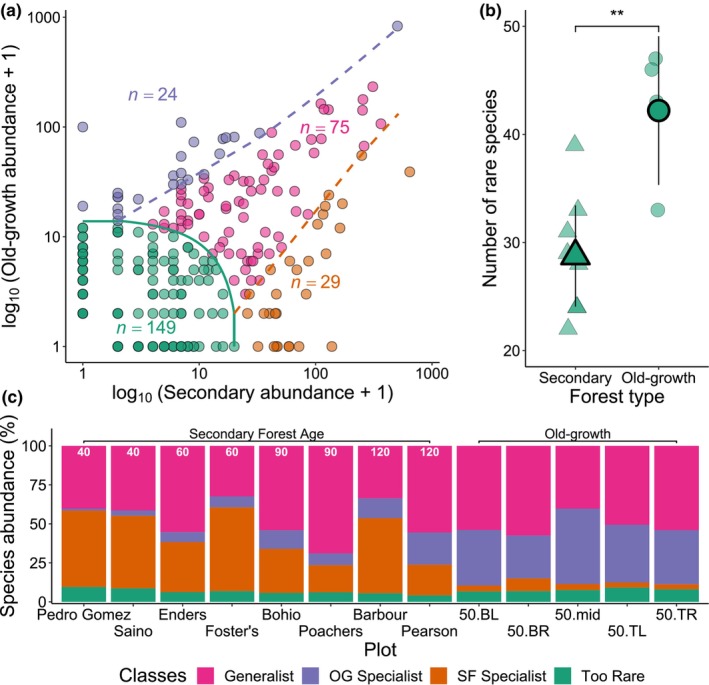
Species classifications from the multinomial model showing: (a). the classification of each species as either a generalist, old‐growth specialist, secondary forest specialist or too rare to classify, according to its abundance in both old‐growth and secondary forest, (b). the number of rare species identified per forest type and (c). the percentage of species in each class per plot (see Table S1 for plot abbreviations). Black outlined shapes indicate the mean ± 95% confidence intervals and jittered points indicate individual plots in Figure 3b.

The multinomial model classified 75 species as generalists, 29 as secondary forest specialists, 24 as old‐growth specialists and 149 as too rare to classify (Figure 3a). As expected, generalist species were common in all plots, and rare species composed a small percentage of total tree abundance (Figure 3c). The proportion of old‐growth specialist species increased through time in the secondary forest plots, indicating gradual floristic convergence (Figure 3c). The number of rare species found in secondary forest plots was significantly lower than in old‐growth plots (*p* = .008; Figure 3b), and this was also found when analyzed over an equal area (Figure S2). Secondary forest plots with low similarity to old‐growth (Figure 2) had a larger proportion of secondary forest specialists and a smaller proportion of generalists than similarly aged secondary plots with higher similarity to old‐growth (Figure 3c).

## 4 DISCUSSION

The chronosequence we study in central Panama is representative of how tropical moist forests regenerate in mid‐to‐late succession, if left undisturbed after agricultural abandonment. Over the timescale of our chronosequence (40–120‐year‐old forests), we find that forest structure, defined as tree stem density and basal area, recovers to old‐growth levels within 90 years (see Denslow, 2000; Denslow & Guzman, 2000; Mascaro et al., 2012). In addition, community composition appears to be converging on old‐growth in one of the 120‐year‐old plots, while the other 120‐year‐old plot appears to be delayed in recovery or following an alternate successional pathway. Old‐growth specialists and rare species did not recover to old‐growth levels of abundance within the timespan of our secondary forest chronosequence. These long timescales for recovery highlight the need to protect secondary forests, as well as existing old‐growth, to facilitate full recovery of forest structure, diversity and composition (Poorter et al., 2021).

We found no relationship between forest structure, richness and diversity and edaphic and topographic variables (tested separately to stand age due to the limited plot sample size), although topography and soil nutrients are known to affect species distributions, community composition and forest structure (Clark et al., 1998; Condit et al., 2013; John et al., 2007; Jucker et al., 2018). It could be that analyzing our plots at a 1‐ha scale, with only 13 plots in total, was insufficient to detect edaphic influences that may occur at more local scales (e.g., Vleminckx et al., 2015). For instance, Phillips et al. (2003) found no edaphic effects on diversity across 10 1‐ha plots but found several edaphic effects when examining 0.1‐ha plots with an equal, or lower, sampling effort. Future work could examine edaphic variables at an intra‐plot level but hereafter we focus the discussion on forest age.

### 4.1 Forest structure, species richness, diversity, and rarity

The results from the expanded plots corroborated previous findings along this chronosequence that forest structure within Panamanian moist secondary forest has fully converged on old‐growth forest by ~90 years. Previous studies within the chronosequence found basal area recovery within 70 years (0.32‐ha plots; Denslow & Guzman, 2000) and aboveground carbon density recovery within 85 years (20‐ha plots; Mascaro et al., 2012). Other aspects of forest structure, including woody debris volume (DeWalt et al., 2003) and large tree/deadwood carbon stocks (Jones et al., 2019), also increase until the later stages of the chronosequence. Our findings are in line with other tropical secondary forests, where forest structural recovery is estimated to take between 27 and 119 years (Poorter et al., 2021).

Species diversity (Simpson's index) and evenness both recovered to old‐growth levels by the start of the chronosequence (40‐years old), with evidence of recovery just 20 years into the chronosequence (Denslow, 2000; Denslow & Guzman, 2000; Dent et al., 2013). This is consistent with previous literature with species diversity recovering within 30 years in Neotropical forest sites in Bolivia (Peña‐Claros, 2003), Colombia and Venezuela (Saldarriaga et al., 1988; Villa et al., 2018). Rapid recovery of species diversity is often linked to the recruitment of both pioneer and shade‐tolerant species early in succession (Finegan, 1996; van Breugel et al., 2007).

In contrast to forest structure and diversity, rarefied species richness in the 1‐ha plots did not show evidence of convergence on old‐growth forest within the timespan of the chronosequence. This conflicts with Dent et al. (2013) who found rapid species richness recovery, within 20 years, when examining midstory and canopy tree species in smaller plots in the 1994 chronosequence census. Differences between the studies remain after excluding understory trees and shrubs to match the Dent et al. (2013) methodology (Figure S6). The larger plot sizes of the current study may have allowed us to detect persistent differences between secondary and old‐growth forests, and increased the likelihood of detecting rarer species with more aggregated distributions (Condit et al., 2000) and species associated with a broader range of edaphic conditions (e.g., John et al., 2007). Subsampled plots less than 0.8 ha in area have been demonstrated to have lower rarefied species richness than 1‐ha plots in tropical forest (Chazdon et al., 2023), and thus the difference between our study and Dent et al. (2013) may largely be one of spatial scale.

The observed lack of species richness recovery contrasts with evidence for median species richness recovery within 54 years across 45 Neotropical secondary forest sites (Rozendaal et al., 2019). However, only one of the 45 sites analyzed included 1‐ha plots and species richness values were rarefied to 25 stems in Rozendaal et al. (2019) compared to 807 stems in our study. Rarefied species richness is also highly sensitive to sample size (Chao & Jost, 2012), and small plots detect fewer rare species (Chazdon et al., 2023), so differences in recovery rates may be methodological (see Figure S3). These results highlight the need to consider plot size and sampling design variation when comparing across studies.

The consistently lower species richness of our secondary forest plots, compared to old‐growth, may be linked to lower numbers of rare species (Figure 3b). On average, secondary forest plots contained 13 fewer rare species than old‐growth plots (Figure 3b), and these species comprise over 50% of the difference in richness between forest types (Figures 1c). This difference could partially be explained by the higher proportion of unidentified species within some secondary compared to old‐growth plots, and thus rare species may be present but not identified in secondary plots (Table S1). However, we still find distinctly fewer rare species in thoroughly identified secondary forest plots and suggest that ecological explanations may be more likely. The paucity of rare species in secondary forest plots is concerning given that rare species can be functionally unique and often contribute disproportionately to regional functional richness (Kearsley et al., 2019; Leitão et al., 2016; Mouillot et al., 2013). The observed lack of rare species even 120 years into succession may indicate that the timescale for full floristic recovery of secondary forests is considerably longer than a century.

A portion of rare tree species may not have yet colonized our secondary forest sites due to dispersal limitation (Arroyo‐Rodríguez et al., 2017; Dent & Estrada‐Villegas, 2021). The BCNM is an intact mosaic of forest where we might expect rapid rates of recovery (Arroyo‐Rodríguez et al., 2017). However the majority of old‐growth forest is located on Barro Colorado Island (Dent & Elsy,  in press), which is physically separated from much of the secondary forest by open water (Figure S1). Proximity to seed sources of old‐growth specialists and rare species may therefore be limited on the peninsulas where five of our secondary forest plots are located. For rare old‐growth species, forests regenerating on peninsulas within the BCNM may experience both source and disperser limitation (Dent & Estrada‐Villegas, 2021) as rare species in the 50‐ha plot on BCI are known to be more aggregated than common species (Condit et al., 2000), and the Gatun Lake potentially acts as a barrier for faunal seed dispersion (Mayhew et al., 2019; Moore et al., 2008). Therefore, the likelihood of rare old‐growth associated species establishing in the secondary forest plots on the peninsulas is likely lower than it would be in a highly connected landscape.

### 4.2 Community composition

Our finding of community composition recovery late in succession mirrors previous work in the chronosequence for lianas (DeWalt et al., 2003) and epiphytes (Woods & DeWalt, 2013), yet differs from previous work on trees which found no clear trend of community composition (Dent et al., 2013). Dent et al. (2013) found that the dominant adult midstory and canopy tree communities (Morisita‐Horn index) of secondary forests were not clearly converging on the composition of old‐growth, but we found evidence of significant recovery, with one 120‐year‐old plot (Pearson) converging on old‐growth levels. Plot size differences between Dent et al. (2013) and our study may partially explain this difference, as larger plots are likely to be more representative of the broader forest stand and disturbances such as treefalls are less likely to influence a larger plot (see Hubbell et al., 1999). We may also be detecting changes that only occurred over the past 20 years (1994 compared to 2014) with most stands becoming more similar to old‐growth forest with time, as tree species typical of old‐growth forest grow into the size range to be inventoried and mid‐successional species and long‐lived pioneers are lost. The latter hypothesis is supported by the increasing proportion of old‐growth specialist species, and decreasing secondary forest specialists, in the older secondary forest sites (Figure 3c). The clear exception to this pattern is the 120‐year‐old Barbour plot, where dominance by *G. superba* appears to be exacerbating differences and leading to an alternate state.

Compositional recovery in a 120‐year‐old secondary forest is an important finding given the extended recovery times predicted for community composition. Median predicted recovery times have been estimated at 487 years to 90% of old‐growth species composition based on chronosequence studies (Rozendaal et al., 2019) or 210 years to 95.5% forest recovery based on pollen records (Cole et al., 2014). In contrast, a large‐scale analysis of tropical secondary forest sites estimated compositional recovery to take 120 years, albeit with much variation among sites (Poorter et al., 2021). Our work, using plots older than typically found in chronosequence comparisons, partially supports the results of Poorter et al. (2021) as we find recovery can occur within 120 years. However, we also show community composition recovery can be highly variable, with the Barbour 120‐year‐old plot far from old‐growth recovery and showing little increase in similarity in the 20 years since the previous census (Dent et al., 2013). Differing land use histories and initial site conditions across the chronosequence may underpin some of the variability in recovery rates (e.g., Estrada‐Villegas et al., 2019; Jakovac et al., 2021), and it could be that remnant trees may have influenced succession in intermediate‐aged BCNM chronosequence stands (Mascaro et al., 2012). However, given the exceptional age of the chronosequence, and the lack of baseline data from secondary forest establishment (Denslow & Guzman, 2000), we were unable to investigate this further.

### 4.3 Evidence for an alternate successional pathway

The 120‐year‐old plot (Barbour) consistently differed from old‐growth and other old secondary forest plots. Barbour had high stem density, low community compositional similarity, was missing old‐growth species, and was dominated by the long‐lived pioneer *G. superba*. This may imply the presence of divergent successional pathways; with a “standard” pathway where structure and composition converge on nearby old‐growth over time, and an “alternate” pathway characterized by early colonization of *G. superba* that delays recovery and alters the species composition.

Early dominance of *G. superba* has been reported in other secondary forests in central Panama (Hooper et al., 2004), and similar evidence of alternate succession can be seen in secondary forests dominated by *Vismia* species on abandoned pastures in the Brazilian Amazon (Mesquita et al., 2015). In our study we see ongoing change in the *G. superba* dominated plot, suggesting that succession is not arrested but slowed; *G. superba* dominance in midstory and canopy trees has decreased by 6% (Table S9) since the 1994 census, when *G. superba* comprised 53% of trees (Dent et al., 2013). The dominance of *G. superba* could be due to multiple interacting mechanisms. Firstly, initial plot conditions may have favored early establishment of *G. superba* post‐disturbance (Estrada‐Villegas et al., 2019; Hooper et al., 2004), and agouti‐mediated feedback loops of seed dispersal in areas of high *G. superba* abundance (Forget, 1992) may have maintained this dominance. Secondly, *G. superba* seeds are resistant to damage and insect infestation (Dalling & Aizprua, 1997; Dalling & Harms, 1999), and *G. superba* can resprout following damage as an adult (Putz & Brokaw, 1989). Resprouting is a key trait linked to monodominance in Amazonia (ter Steege et al., 2019), and thus these traits likely maintain high *G. superba* abundance. Our finding of high species dominance throughout succession links to evidence of monodominance persistence for long time periods in many biogeographic regions (Anbarashan & Parthasarathy, 2013; Hart et al., 1989; ter Steege et al., 2019) and provides evidence that high species dominance in secondary forests (e.g., Longworth et al., 2014; Oberleitner et al., 2021) can persist past the early stages of succession.

### 4.4 Ongoing differences between old‐growth and secondary forests

There were clear differences in species composition between old‐growth and secondary forest plots, despite the secondary forest maturity. In the BCNM secondary forests, the number of rare species had not recovered to old‐growth values, even though overall rare tree abundance was similar between plots (Figure 3c), and old‐growth specialist species were still increasing in abundance in older secondary forests, including in the 120‐year‐old plots (Figure 3c). The gradual recovery of old‐growth specialists could be due to the long lifespans of many long‐lived pioneers. Even relatively shade‐intolerant species can live for c. 150 years in wet tropical forest (Köhl et al., 2017), and long‐lived pioneers are known to persist late into succession, with some species living for up to 200 years (Vlam et al., 2017). Establishment of old‐growth specialists late in succession may also be linked to more conservative resource acquisition strategies that lead to old‐growth specialists being better competitors in mature forests (e.g., Boukili & Chazdon (2017) but see Letcher et al. (2015)). Further work investigating if our species classifications are associated with different functional traits would allow us to better understand how the slow recovery of old‐growth specialists and rare species relates to forest function.

Ongoing differences between secondary and old‐growth forests may partly be due to spatial aggregation of old‐growth plots compared to the secondary forest plots. A portion of the old‐growth specialists and rare species that we identified may be associated with local‐scale topographic or edaphic conditions (John et al., 2007; Jucker et al., 2018; Toledo et al., 2012) within the 50‐ha forest dynamics plot, rather than wider landscape of which the secondary forest plots are more representative. This is certainly possible given that we see evidence of spatial autocorrelation between plot location and environmental variables (see Supporting Information). However, the broad spatial configuration of secondary forest plots within the study landscape likely provides more heterogeneous environmental conditions than occur within the old‐growth plots, which can be related to higher species diversity (Xu et al., 2016). Despite this, we see higher numbers of rare species in old‐growth compared to secondary forests, suggesting that forest age strongly influences rare species occurrence. More generally, old‐growth forests typically comprise larger forest fragments in tropical landscapes (Hansen et al., 2020) and we believe the configuration of secondary and old‐growth forest plots across the BCNM is representative of fragmented forest landscapes across the Neotropics.

### 4.5 Conclusions

Our study in the BCNM secondary forest chronosequence demonstrates that forest structure can recover within c. 90 years, species diversity (but not species richness) in <40 years, and community composition by 120 years. We find further recovery in community composition in the 20‐years since the previous chronosequence census but also find differing species richness results (Dent et al., 2013), emphasizing the importance of both temporal and spatial scale in examining secondary forest recovery. Divergent successional pathways were also found within the chronosequence, with one of the 120‐year‐old‐plots following an alternate pathway of high species dominance. Despite these recovery patterns, we find evidence that full old‐growth convergence is unlikely over the timescale of our chronosequence, as rare species were missing from secondary forest plots, and old‐growth specialists were in low abundance. Given the long time frames involved in community composition recovery, it is imperative that existing old‐growth and old secondary forests are protected to safeguard unique tree communities and contribute to global biodiversity conservation targets under the Kunming‐Montreal Global Biodiversity Framework (Convention on Biological Diversity, 2022).

## Supporting information


**Data S1:** Supporting Information

## Data Availability

The data that support the findings of this study are openly available in the Dryad Digital Repository: https://doi.org/10.5061/dryad.02v6wwq8x (Elsy et al., 2023).
