# Supplementary material for: Incomplete recovery of tree community composition and rare species after 120 years of tropical forest succession in Panama
Source: Biotropica. 2023 Oct 30;56(1):36–49. doi: 10.1111/btp.13275 (PMC10952663; doi:10.1111/btp.13275)
Supplement: Supplementary file 1 — Data S1: Supporting Information [file BTP-56-36-s001.docx]

SUPPORTING INFORMATION

**Incomplete recovery of tree community composition and rare species after 120 years of tropical forest succession in Panama**

Alexander D. Elsy^1^, Marion Pfeifer^2^, Isabel L. Jones^1^, Saara J. DeWalt^3^, Omar R. Lopez^4,5^, Daisy H. Dent^4,6,7^

1. Biological and Environmental Sciences, University of Stirling, Stirling, FK9 4LA, UK.
2. School of Natural and Environmental Sciences, Modelling, Evidence and Policy Group, Newcastle University, Newcastle Upon Tyne NE1 7RU, UK
3. Department of Biological Sciences, Clemson University, Clemson, SC USA
4. Smithsonian Tropical Research Institute, Apartado 0843–03092, Balboa, Panama
5. Instituto de Investigaciones Científicas y Servicios de Alta Tecnología (INDICASAT), Edificio 209, Clayton, Panama.
6. Max Planck Institute for Animal Behavior, Konstanz, Germany
7. Department of Environmental Systems Science, ETH Zürich, Zurich, Switzerland.

Appendix S1.

**METHODS:**

**Study area:**

The 1-ha plots were positioned to overlap the previous 0.32-ha plots where possible and all 1-ha plots are in the same forest stands, and same aged forest, as the 0.32-ha plots. However, one 40-year-old plot (Pedro Gomez, Table S1) and one 60-year-old plot (Foster’s, Table S1) do not directly overlap with the 1994 0.32-ha plots due to constraints when laying out the 1-ha plots.

**Data exploration:**

Environmental variables (stand age, mean soil nitrogen, mean soil phosphorous and mean slope) were tested for collinearity prior to analysis and no evidence of collinearity was found (all variance inflation factors < 5). There was evidence of spatial autocorrelation between N, P and slope variables and plot location (tested using Moran’s *I*). However, there was no evidence of stand age being autocorrelated with plot location, when a 500-year-old proxy age was assigned to old-growth plots. Each explanatory variable was centred and scaled by dividing by two standard deviations before analysis using the ‘rescale’ function (Gelman & Su, 2022; Schielzeth, 2010).

Additional data exploration was carried out with the tree census data to allow a direct comparison with Dent et al. (2013). All trees, palms and shrubs ≥ 5cm DBH were subset, in a separate analysis, to only tree or palm species which reached the midstory (maximum height = 10 – 30 m) or canopy (maximum height > 30 m) to allow the recovery of dominant species to be quantified between studies (Dent et al., 2013; Table S9). Rarefied species richness (rarefied to the same number of stems as Dent et al. (2013); n = 120), Pielou’s evenness and Simpson’s diversity indices were also calculated for this subset (Figure S6).

**Model fitting:**

All diversity, forest structure and community composition data were modelled using either generalised linear models (GLMs) or beta regressions against plot age, mean soil nitrogen and phosphorous, and mean plot slope. The choice of model for each response variable can be seen in Table S2. We determined the distribution required for each model based on the underlying assumptions of each family, residual and QQ plots, and tests of normality, dispersion and outliers generated through the ‘simulateResiduals’ function in the ‘DHARMa’ R package (Hartig, 2022). Negative binomial GLMs were used when count response variables were overdispersed, or residual fit improved through their usage. Rarefied richness results were rounded before analysis, to enable them to be modelled as count data. R-squared values for each model were computed using the ‘r2’ function from the ‘performance’ R package (Lüdecke et al., 2021). Pielou’s evenness index and Simpson’s diversity index were modelled using beta regressions from the ‘betareg’ R package (Cribari-Neto & Zeileis, 2010). These data fit the beta distribution as the values were continuous and bounded between 0 and 1 (Cribari-Neto & Zeileis, 2010). Beta regressions were run with a logit link and fixed precision parameters (with an identity link).

| **Table S1**. Summary statistics for all trees, palms, and shrubs ≥ 5 cm in each 1-ha plot in the Barro Colorado Nature Monument (sampled between 2011 and 2016). Environmental variables were averaged per 1-ha plot with soil data obtained from Jones et al. (2019) and Wolf et al. (2015) and topographic data from the Smithsonian Tropical Research Institute (2020). Land use history information was obtained from Denslow and Guzman (2000)**.** Old-growth plot codes refer to the location from which they were subsampled from in the 50-ha plot (i.e., 50.TL is the 1-ha plot from the top left of the 50-ha plot). | | | | | | | | | | |
| --- | --- | --- | --- | --- | --- | --- | --- | --- | --- | --- |
| **Plot (code)** | **Age** | **Location** | **Land use history** | **Bedrock^a^** | **No. of individuals (*no. stems*)** | **Percent identified^b^** | **Species richness** | **N (%)** | **P (mg kg^-1^)** | **Slope (**^o^**)** |
| **Saino** (SAI) | 40 | Gigante | Pasture/swidden | Basalt | 943 (*1214*) | 95.798 | 96 | 0.517 | 3.174 | 8.800 |
| **Pedro Gomez** (PED) | 40 | Gigante | Pasture/swidden | Basalt | 933 (*1053*) | 99.005 | 106 | 0.373 | 3.697 | 3.994 |
| **Foster’s** (FOS) | 60 | Gigante | Plantation | Basalt | 1148 (*1404*) | 99.130 | 125 | 0.689 | 7.276 | 4.613 |
| **Enders** (END) | 60 | Gigante | Pasture/swidden | Basalt | 1042 (*1242*) | 98.560 | 126 | 0.428 | 3.958 | 9.371 |
| **Bohio** (BOH) | 90 | Bohio | Pasture/swidden | Bohio | 808 (875) | 99.876 | 95 | 0.431 | 7.271 | 16.236 |
| **Poachers** (POA) | 90 | BCI | Pasture | Caimito marine | 1056 (*1167*) | 94.883 | 108 | 0.375 | 7.234 | 8.240 |
| **Pearson** (PEA) | 120 | BCI | Pasture | Bohio | 820 (*987*) | 99.878 | 114 | 0.461 | 7.200 | 7.554 |
| **Barbour** (BAR) | 120 | BCI | Pasture | Caimito volcanic | 1082 (*1427*) | 99.445 | 84 | 0.369 | 3.783 | 3.321 |
| **50.TL** | OG | BCI | Old-growth | Andesite | 1050 (*1178*) | 100.0 | 136 | 0.453 | 2.255 | 8.462 |
| **50.BL** | OG | BCI | Old-growth | Andesite | 1094 (*1275*) | 100.0 | 135 | 0.506 | 2.660 | 8.151 |
| **50.mid** | OG | BCI | Old-growth | Andesite | 1024 (*1175*) | 100.0 | 133 | 0.453 | 4.080 | 4.777 |
| **50.TR** | OG | BCI | Old-growth | Andesite | 1055 (*1170*) | 100.0 | 148 | 0.400 | 1.826 | 6.875 |
| **50.BR** | OG | BCI | Old-growth | Andesite | 918 (*1130*) | 100.0 | 122 | 0.532 | 2.189 | 4.909 |
| ^a^ Baillie et al., (2006), Denslow & Guzman (2000), ^b^ Percent identified refers to the percentage of individual trees identified to a species level in each plot. | | | | | | | | | | |


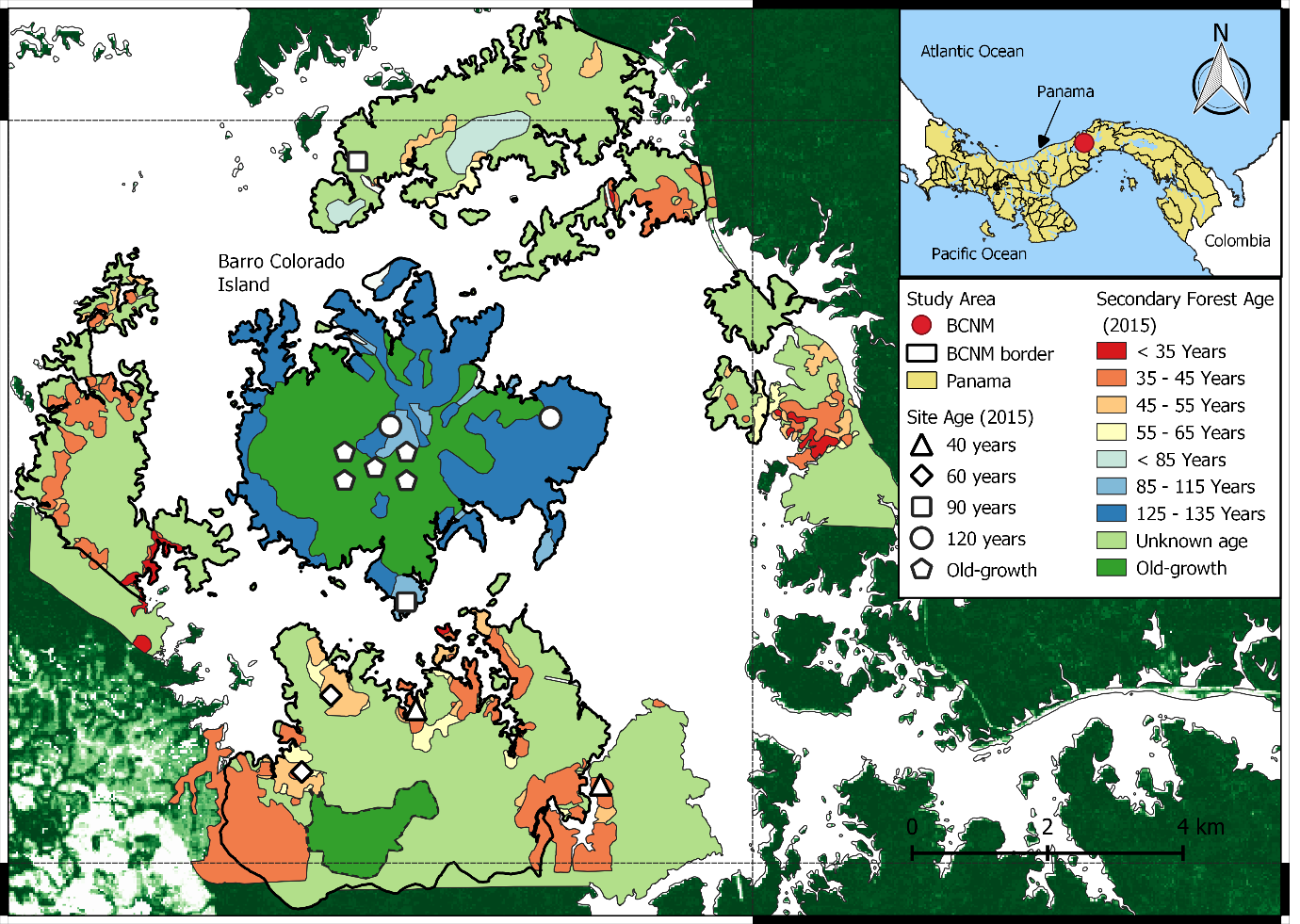


**Figure S1**. Map displaying study plots around the Barro Colorado Nature Monument (BCNM), central Panama. White shapes show 1-ha forest plots, with shape determined by stand age at time of census (2011-2016). Approximate secondary forest stand ages (as of 2015) and old growth forest extents were obtained from Dent and Elsy (*in press*). Forest ages were informed by Enders (1935), Kenoyer (1929), and aerial photographs taken by the US defence mapping agency (in 1955, 1966, 1973, 1979 and 1983). The red circle, on the country scale map, indicates the location of the BCNM within Panama. Country shapefiles were obtained from Global Administrative Areas (Hijmans et al., 2020) and the BCNM shapefiles are from the Smithsonian Tropical Research Institute Data Portal (2019). The map was made using QGIS v.3.12.3 (QGIS Development Team, 2020).

| **Table S2.** Model specifications used to model both the effect of stand age on secondary forest structural and diversity metrics and to model the influence of environmental variables in all plots. Environmental variable models (mean N, mean P, and mean slope) are full models specified prior to model selection. | | | |
| --- | --- | --- | --- |
| **Secondary forest plots (*n* = 8)** | | | |
| **Model specification** | **Distribution** | **Link function** | ***R* function (package)** |
| Stem Density ~ Stand Age | Negative binomial | log | glm.nb (MASS) |
| Basal Area ~ Stand Age | Gaussian | identity | glm (stats) |
| Rarefied Species Richness ~ Stand Age | Poisson | log | glm (stats) |
| Simpson’s Diversity Index ~ Stand Age | Beta | logit | betareg (betareg) |
| Pielou’s Evenness Index ~ Stand Age | Beta | logit | betareg (betareg) |
| **All plots (*n* = 13)** | | | |
| **Full model specification** | **Distribution** | **Link function** | ***R* function (package)** |
| Stem Density ~ P + N + Slope | Negative binomial | log | glm.nb (MASS) |
| Basal Area ~ P + N + Slope | Gaussian | identity | glm (stats) |
| Rarefied Species Richness ~ P + N + Slope | Negative binomial | log | glm.nb (MASS) |
| Simpson’s Diversity Index ~ P + N + Slope | Beta | logit | betareg (betareg) |
| Pielou’s Evenness Index ~ P + N + Slope | Beta | logit | betareg (betareg) |

| **Table S3.** Generalised Linear Model and beta regression results examining the effect of age on secondary forest structure and diversity metrics. Both the untransformed coefficients (second column) and exponentiated coefficients are reported where appropriate. | | | | | | | | |
| --- | --- | --- | --- | --- | --- | --- | --- | --- |
| **Stem Density** | | | | | | | | |
| **Predictors** | **Log Mean (CI 95%)** | **Incidence Rate Ratios (CI 95%)** | | | **Statistic** | | ***P value*** | ***R^2^*** |
| **(Intercept)** | 7.058 (6.787 – 7.335) | 1161.984 (886.642 – 1533.426) | | | 50.119 | | < 0.001 | 0.006 |
| **Stand age** | 0.000 (-0.003– 0.004) | 1.000 (0.997 – 1.004) | | | 0.169 | | 0.886 |  |
| **Basal Area** | | | | | | | | |
| **Predictors** | **Estimates (CI 95%)** | | **Statistic** | ***P value*** | | ***R^2^*** | | |
| **(Intercept)** | 16.742 (14.592 – 18.892) | | 15.263 | < 0.001 | | 0.911 | | |
| **Stand age** | 0.103 (0.077 – 0.129) | | 7.815 | <0.001 | |  |  |  |
| **Rarefied Species Richness** | | | | | | | | |
| **Predictors** | **Log Mean (CI 95%)** | **Incidence Rate Ratios (CI 95%)** | | | **Statistic** | | ***P value*** | ***R^2^*** |
| **(Intercept)** | 4.703 (4.513 – 4.889) | 110.244 (91.206 – 132.789) | | | 49.085 | | < 0.001 | 0.148 |
| **Stand age** | -0.001 (-0.003 – 0.001) | 0.999 (0.997 – 1.001) | | | -0.990 | | 0.322 |  |
| **Simpson’s Diversity Index** | | | | | | | | |
| **Predictors** | **Estimate (CI 95%)** | **Exp (Estimate (CI 95 %))** | | | **Statistic** | | ***P value*** | ***R^2^*** |
| **(Intercept)** | 3.628 (2.662 – 4.594) | 37.643 (14.331 – 98.881) | | | 7.363 | | < 0.001 | 0.323 |
| **Stand age** | -0.010 (-0.020 – 0.001) | 0.991 (0.980 – 1.001) | | | -1.762 | | 0.078 |  |
| **Dispersion (*phi*)** | 81.06 (0.484 - 161.636) | - | | | 1.972 | | 0.0486 |  |
| **Pielou’s Evenness Index** | | | | | | | | |
| **Predictors** | **Estimate (CI 95%)** | **Exp (Estimate (CI 95 %))** | | | **Statistic** | | ***P value*** | ***R^2^*** |
| **(Intercept)** | 1.816 (1.322 – 2.309) | 6.145 (3.753 – 10.063) | | | 7.215 | | < 0.001 | 0.397 |
| **Stand age** | -0.006 (-0.012 – -0.001) | 0.994 (0.988 – 0.999) | | | -2.201 | | 0.028 |  |
| **Dispersion (*phi*)** | 93.65 (2.18 – 185.12) | - | | | 2.007 | | 0.0448 |  |

| **Table S4.** Variables selected within Δ AICc ≤ 4 for models of structural and diversity metrics using all plots. Model weights do not sum to 1 due to the exclusion of models with Δ AICc > 4. Note *R^2^* values cannot currently be computed by MuMIN for beta regression model selection (Bartoń, 2022) and so are excluded for: Simpson’s diversity index and Pielou’s evenness index. | | | | |
| --- | --- | --- | --- | --- |
| **Variables included in model** | **AICc** | **Δ AICc** | **Weight** | **Adjusted *R^2^*** |
| **STEM DENSITY:** |  |  |  |  |
| **Intercept + mean slope** | 167.409 | 0.000 | 0.577 | 0.397 |
| **Intercept + mean N + mean slope** | 170.318 | 2.809 | 0.142 | 0.464 |
| **Intercept (only)** | 170.529 | 3.120 | 0.121 | 0.000 |
| **Intercept + mean P + mean slope** | 171.364 | 3.955 | 0.080 | 0.415 |
| **BASAL AREA:** |  |  |  |  |
| **Intercept (only)** | 75.122 | 0.000 | 0.550 | 0.000 |
| **Intercept + mean N** | 77.341 | 2.220 | 0.181 | 0.092 |
| **Intercept + mean P** | 78.387 | 3.265 | 0.108 | 0.015 |
| **Intercept + mean slope** | 78.530 | 3.408 | 0.100 | 0.005 |
| **RAREFIED SPECIES RICHNESS:** |  |  |  |  |
| **Intercept (only)** | 114.031 | 0.000 | 0.479 | 0.000 |
| **Intercept + mean P** | 115.543 | 1.512 | 0.225 | 0.140 |
| **Intercept + mean N** | 116.848 | 2.818 | 0.117 | 0.049 |
| **Intercept + mean slope** | 117.461 | 3.430 | 0.086 | 0.003 |
| **SIMPSON’S DIVERSITY INDEX:** |  |  |  |  |
| **Intercept (only)** | -52.191 | 0.000 | 0.500 | - |
| **Intercept + mean N** | -50.660 | 1.530 | 0.232 | - |
| **Intercept + mean slope** | -48.944 | 3.246 | 0.099 | - |
| **Intercept + mean P** | -48.724 | 3.467 | 0.088 | - |
| **PIELOU’S EVENNESS INDEX:** |  |  |  |  |
| **Intercept (only)** | -34.812 | 0.000 | 0.488 | - |
| **Intercept + mean N** | -32.935 | 1.877 | 0.191 | - |
| **Intercept + mean slope** | -32.190 | 2.622 | 0.131 | - |
| **Intercept + mean P** | -31.349 | 3.464 | 0.086 | - |

| **Table S5.** Full model-averaged parameter estimates for models of structural and diversity metrics with AICc ≤ 4. Estimates are for centred and rescaled (divided by two standard deviations) explanatory variables. | | | | |
| --- | --- | --- | --- | --- |
| **Predictors** | **Estimate** | **Adjusted standard error** | ***z* statistic** | ***P* value** |
| **STEM DENSITY:** |  |  |  |  |
| **Intercept** | 7.086 | 0.026 | 241.593 | < 0.001 |
| **Mean slope** | -0.139 | 0.073 | 1.795 | 0.073 |
| **Mean N** | 0.010 | 0.030 | 0.305 | 0.761 |
| **Mean P** | 0.003 | 0.019 | 0.141 | 0.888 |
| **BASAL AREA:** |  |  |  |  |
| **Intercept** | 26.055 | 1.036 | 22.516 | < 0.001 |
| **Mean N** | -0.433 | 1.289 | 0.315 | 0.753 |
| **Mean P** | 0.105 | 0.806 | 0.118 | 0.906 |
| **Mean slope** | 0.053 | 0.744 | 0.064 | 0.949 |
| **RAREFIED SPECIES RICHNESS:** |  |  |  |  |
| **Intercept** | 4.701 | 0.044 | 107.735 | < 0.001 |
| **Mean P** | -0.028 | 0.065 | 0.427 | 0.669 |
| **Mean N** | 0.009 | 0.039 | 0.219 | 0.827 |
| **Mean slope** | -0.002 | 0.029 | 0.053 | 0.958 |
| **SIMPSON’S DIVERSITY INDEX:** |  |  |  |  |
| **Intercept** | 2.896 | 0.183 | 15.823 | < 0.001 |
| **Dispersion (*phi*)** | 58.790 | 27.006 | 2.177 | 0.030 |
| **Mean slope** | 0.135 | 0.301 | 0.449 | 0.654 |
| **Mean P** | -0.015 | 0.124 | 0.120 | 0.904 |
| **Mean N** | -0.000 | 0.112 | 0.003 | 0.997 |
| **PIELOU’S EVENNESS INDEX:** |  |  |  |  |
| **Intercept** | 1.362 | 0.100 | 13.647 | < 0.001 |
| **Dispersion (*phi*)** | 59.852 | 26.612 | 2.249 | 0.025 |
| **Mean slope** | 0.052 | 0.139 | 0.376 | 0.707 |
| **Mean P** | -0.024 | 0.097 | 0.248 | 0.804 |
| **Mean N** | 0.001 | 0.066 | 0.015 | 0.988 |

| **Table S6.** Outputs of two-sample Wilcoxon tests investigating the difference between secondary and old-growth forest for forest structure and diversity metrics. | | | |
| --- | --- | --- | --- |
| **Metric** | **Comparison** | **Statistic** | ***P* value** |
| **Stem Density** | Old-growth vs Secondary | 20 | 1.000 |
| **Basal Area** | Old-growth vs Secondary | 30 | 0.171 |
| **Rarefied Species Richness** | Old-growth vs Secondary | 40 | 0.002 |
| **Extrapolated Species Richness** | Old-growth vs Secondary | 37 | 0.011 |
| **Simpson’s Diversity Index** | Old-growth vs Secondary | 28 | 0.284 |
| **Pielou’s Evenness Index** | Old-growth vs Secondary | 26 | 0.435 |
| **Number of Rare Species** | Old-growth vs Secondary | 38.5 | 0.008 |

| **Table S7.** Generalised Linear Mixed Model results for pairwise comparisons of Sørensen, Horn and Morisita-Horn indices of similarity between secondary forest and old-growth plots. All GLMMs were run with a beta distribution and with plot as a random effect (eight levels, five replicates per level) with a random intercept. Both the untransformed coefficients (second column) and exponentiated coefficients are reported. Conditional *R^2^* is not reported due to the uncertainty in the random effect variance. | | | | | |
| --- | --- | --- | --- | --- | --- |
| **Sørensen Similarity Index** | | | | | |
| **Predictors** | **Estimate (CI 95%)** | **Exp(Estimate (CI 95%))** | **Statistic** | ***P value*** | ***Marginal R^2^*** |
| **(Intercept)** | 0.325 (-0.342 –1.013) | 1.399 (0.711 – 2.753) | 0.971 | 0.322 | 0.293 |
| **Stand age** | 0.006 (-0.002 – 0.014) | 1.006 (0.998– 1.014) | 1.461 | 0.144 |  |
| **Dispersion (*phi*)** | 33.682 (20.729 – 54.725) | - | - | - |  |
| **Random effect variance** = 0.101 | |  |  |  |  |
| **Horn Similarity Index** | | | | | |
| **Predictors** | **Estimate (CI 95%)** | **Exp(Estimate (CI 95%))** | **Statistic** | ***P value*** | ***Marginal R^2^*** |
| **(Intercept)** | -0.294 (-0.992 – 0.403) | 0.745 (0.371 – 1.496) | -0.827 | 0.408 | 0.467 |
| **Stand age** | 0.011 (0.002 – 0.019) | 1.011 (1.002 – 1.019) | 2.485 | 0.013 |  |
| **Dispersion (*phi*)** | 102.264 (62.783 – 166.574) | - | - | - |  |
| **Random effect variance** = 0.126 | |  |  |  |  |
| **Morisita-Horn Similarity Index** | | | | | |
| **Predictors** | **Estimate (CI 95%)** | **Exp(Estimate (CI 95%))** | **Statistic** | ***P value*** | ***Marginal R^2^*** |
| **(Intercept)** | -1.607 (-2.826 – -0.387) | 0.201 (0.059 – 0.679) | -2.582 | 0.010 | 0.389 |
| **Stand age** | 0.016 (0.002 – 0.031) | 1.016 (1.002 – 1.031) | 2.190 | 0.029 |  |
| **Dispersion (*phi*)** | 41.904 (25.819 – 68.010) | - | - | - |  |
| **Random effect variance** = 0.386 | |  |  |  |  |


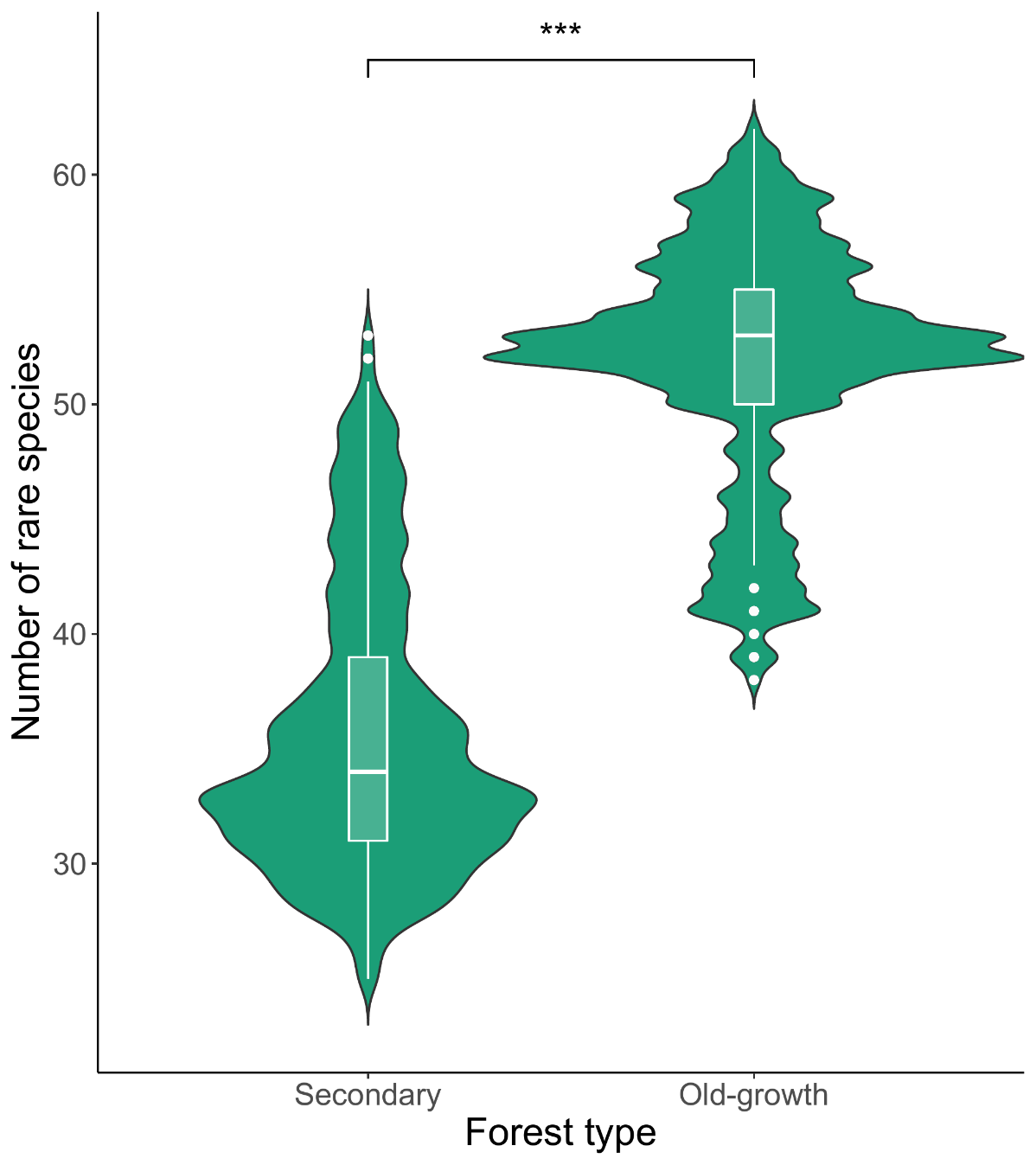


**Figure S2.** The number of locally rare species identified in either secondary or old-growth forest by the multinomial model for all possible combinations of the comparison between five 1-ha secondary forest plots (*n* = 6,720) and the five 1-ha old-growth plots. The asterisks indicate results of a two-tailed *t-*test between forest type (*P* < 0.001). Violin plots indicate the data distribution, and the box plots demonstrate the median, interquartile range, and outliers.


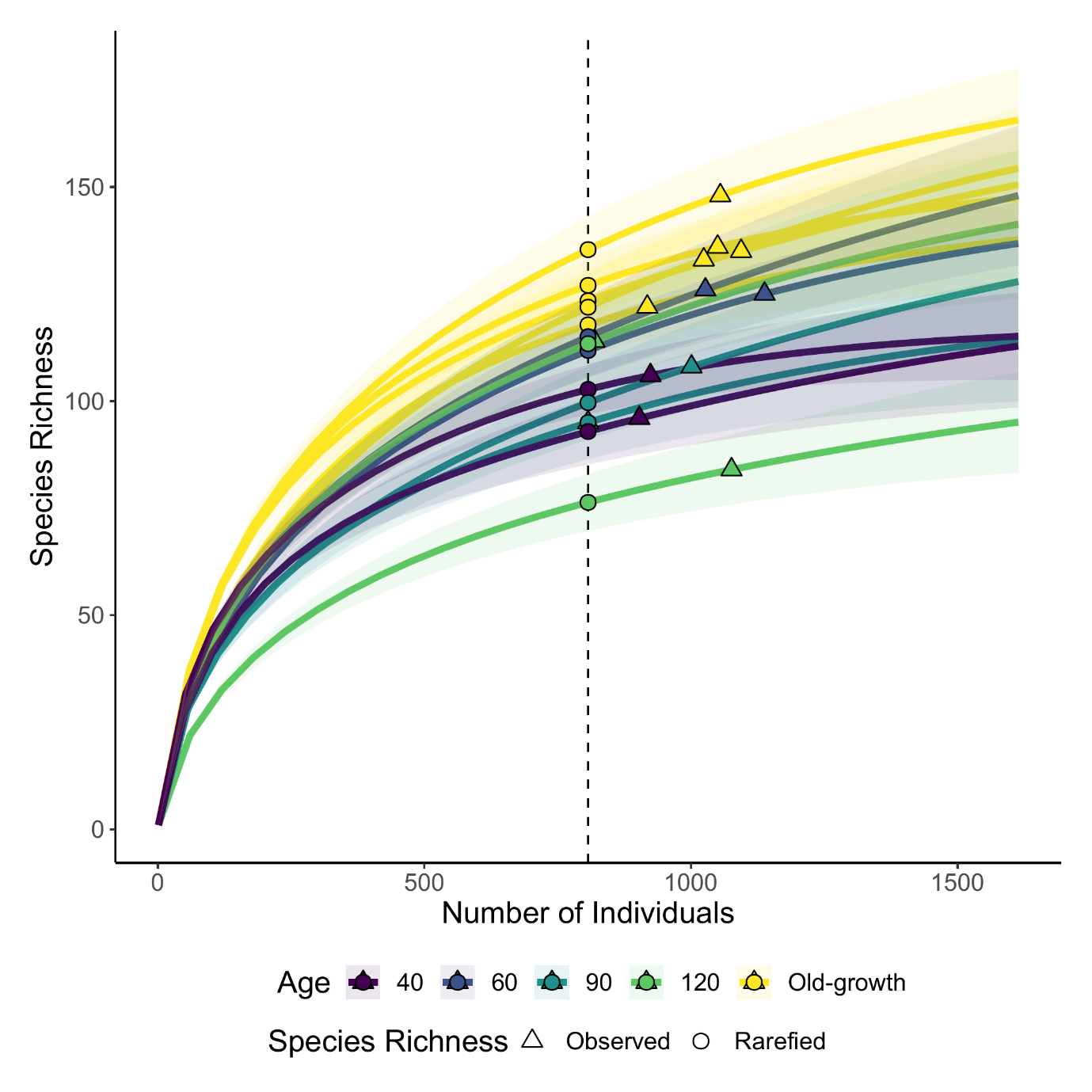


**Figure S3.** Species rarefaction/extrapolation curves displaying observed, rarefied, and extrapolated species richness. The dashed line indicates the number of individuals to which species richness values were rarefied to, i.e. the minimum number of individual identified trees found per plot (*n* = 807). Shading either side of the line represents 95 % confidence intervals for rarefaction and extrapolation predictions.

**Table S8**. The five most abundant tree, palm or shrub species ≥ 5 cm per 1-ha plot.

| Age | Plot | Species | Number of individuals | Percent of total individuals (%) |
| --- | --- | --- | --- | --- |
| 40 years | **Pedro Gomez** | *Cupania seemannii* | 77 | 8.33 |
|  |  | *Malouetia guatemalensis* | 58 | 6.28 |
|  |  | *Lacistema aggregatum* | 41 | 4.44 |
|  |  | *Xylopia frutescens* | 35 | 3.79 |
|  |  | *Protium panamense* | 34 | 3.68 |
| 40 years | **Saino** | *Swartzia simplex* | 126 | 13.95 |
|  |  | *Gustavia superba* | 74 | 8.19 |
|  |  | *Miconia argentea* | 56 | 6.20 |
|  |  | *Inga sapindoides* | 55 | 6.09 |
|  |  | *Spondias radlkoferi* | 43 | 4.76 |
| 60 years | **Enders** | *Oenocarpus mapora* | 116 | 11.30 |
|  |  | *Gustavia superba* | 60 | 5.84 |
|  |  | *Mabea occidentalis* | 52 | 5.06 |
|  |  | *Alseis blackiana* | 46 | 4.48 |
|  |  | *Trichilia tuberculata* | 46 | 4.48 |
| 60 years | **Foster's** | *Coussarea curvigemma* | 215 | 18.89 |
|  |  | *Faramea occidentalis* | 67 | 5.89 |
|  |  | *Inga vera* | 58 | 5.10 |
|  |  | *Alseis blackiana* | 50 | 4.39 |
|  |  | *Myrciaria floribunda* | 43 | 3.78 |
| 90 years | **Bohio** | *Faramea occidentalis* | 78 | 9.67 |
|  |  | *Cupania seemannii* | 58 | 7.19 |
|  |  | *Gustavia superba* | 53 | 6.57 |
|  |  | *Virola sebifera* | 47 | 5.82 |
|  |  | *Maquira guianensis* | 45 | 5.58 |
| 90 years | **Poachers** | *Trichilia tuberculata* | 106 | 10.59 |
|  |  | *Protium panamense* | 92 | 9.19 |
|  |  | *Swartzia simplex* | 68 | 6.79 |
|  |  | *Oenocarpus mapora* | 55 | 5.49 |
|  |  | *Xylopia macrantha* | 50 | 5.00 |
| 120 years | **Barbour** | *Gustavia superba* | 384 | 35.69 |
|  |  | *Faramea occidentalis* | 124 | 11.52 |
|  |  | *Oenocarpus mapora* | 58 | 5.39 |
|  |  | *Swartzia simplex* | 58 | 5.39 |
|  |  | *Alseis blackiana* | 34 | 3.16 |
| 120 years | **Pearson** | *Faramea occidentalis* | 110 | 13.43 |
|  |  | *Trichilia tuberculata* | 63 | 7.69 |
|  |  | *Oenocarpus mapora* | 48 | 5.86 |
|  |  | *Astrocaryum standleyanum* | 43 | 5.25 |
|  |  | *Swartzia simplex* | 43 | 5.25 |
| Old-growth | **50-ha BL** | *Faramea occidentalis* | 147 | 13.44 |
|  |  | *Alseis blackiana* | 49 | 4.48 |
|  |  | *Oenocarpus mapora* | 46 | 4.20 |
|  |  | *Desmopsis panamensis* | 40 | 3.66 |
|  |  | *Prioria copaifera* | 27 | 2.47 |
| Old-growth | **50-ha BR** | *Faramea occidentalis* | 121 | 13.18 |
|  |  | *Oenocarpus mapora* | 72 | 7.84 |
|  |  | *Drypetes standleyi* | 53 | 5.77 |
|  |  | *Alseis blackiana* | 51 | 5.56 |
|  |  | *Hirtella triandra* | 48 | 5.23 |
| Old-growth | **50-ha Mid** | *Faramea occidentalis* | 338 | 33.01 |
|  |  | *Trichilia tuberculata* | 48 | 4.69 |
|  |  | *Oenocarpus mapora* | 35 | 3.42 |
|  |  | *Tetragastris panamensis* | 35 | 3.42 |
|  |  | *Swartzia simplex* | 31 | 3.03 |
| Old-growth | **50-ha TL** | *Faramea occidentalis* | 148 | 14.10 |
|  |  | *Socratea exorrhiza* | 57 | 5.43 |
|  |  | *Alseis blackiana* | 50 | 4.76 |
|  |  | *Oenocarpus mapora* | 40 | 3.81 |
|  |  | *Tetragastris panamensis* | 26 | 2.48 |
| Old-growth | **50-ha TR** | *Faramea occidentalis* | 76 | 7.20 |
|  |  | *Hirtella triandra* | 55 | 5.21 |
|  |  | *Protium tenuifolium* | 55 | 5.21 |
|  |  | *Drypetes standleyi* | 43 | 4.08 |
|  |  | *Oenocarpus mapora* | 39 | 3.70 |


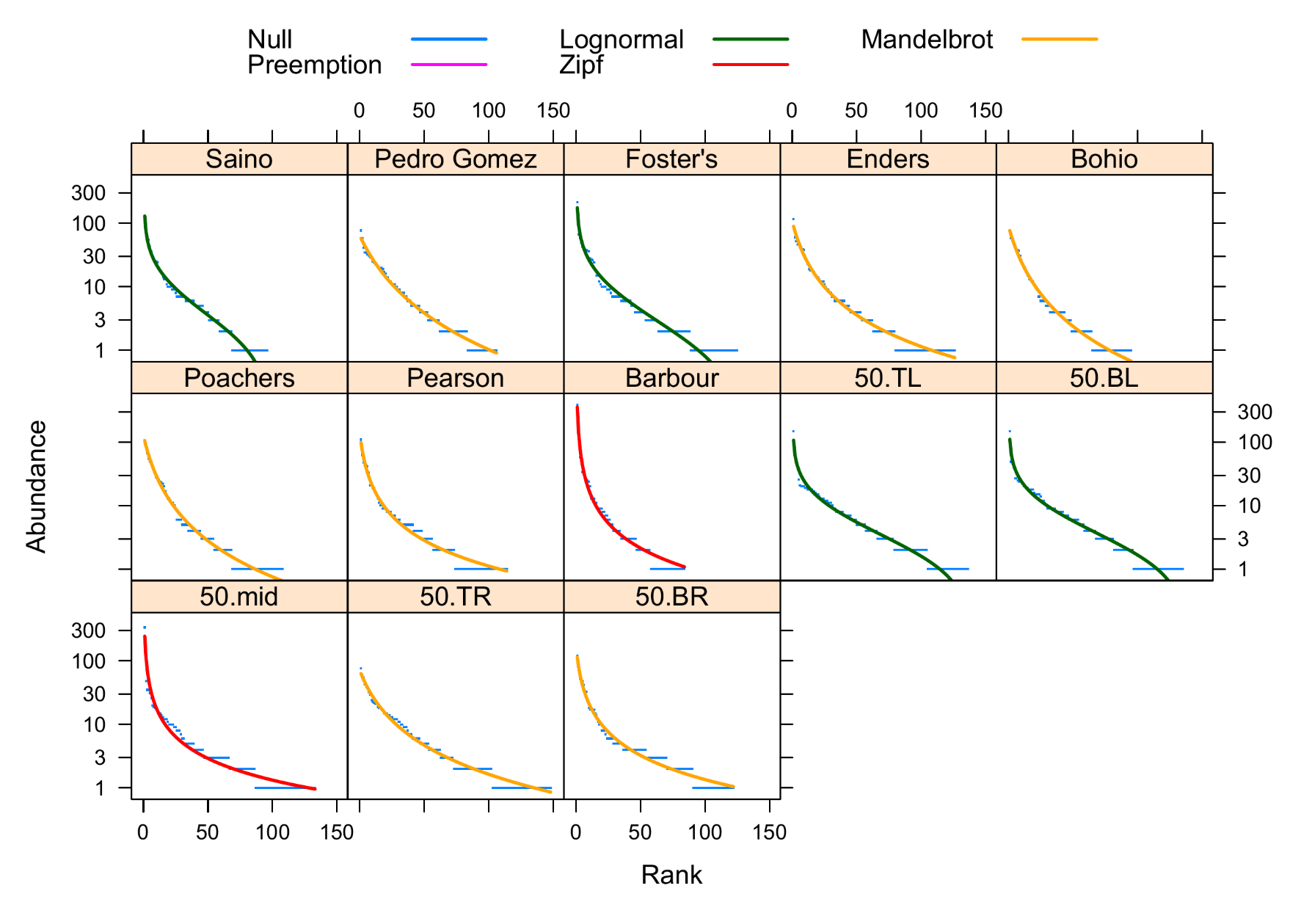


**Figure S4.** Rank abundance plot demonstrating the differences in species dominance among plots. Log species abundance is plotted against species ranked by abundance using the radfit function in ‘vegan’ (Oksanen et al., 2022). Different coloured lines correspond to the best fitting rank abundance model (all with Poisson errors) based on AIC and an automatic fitting procedure.


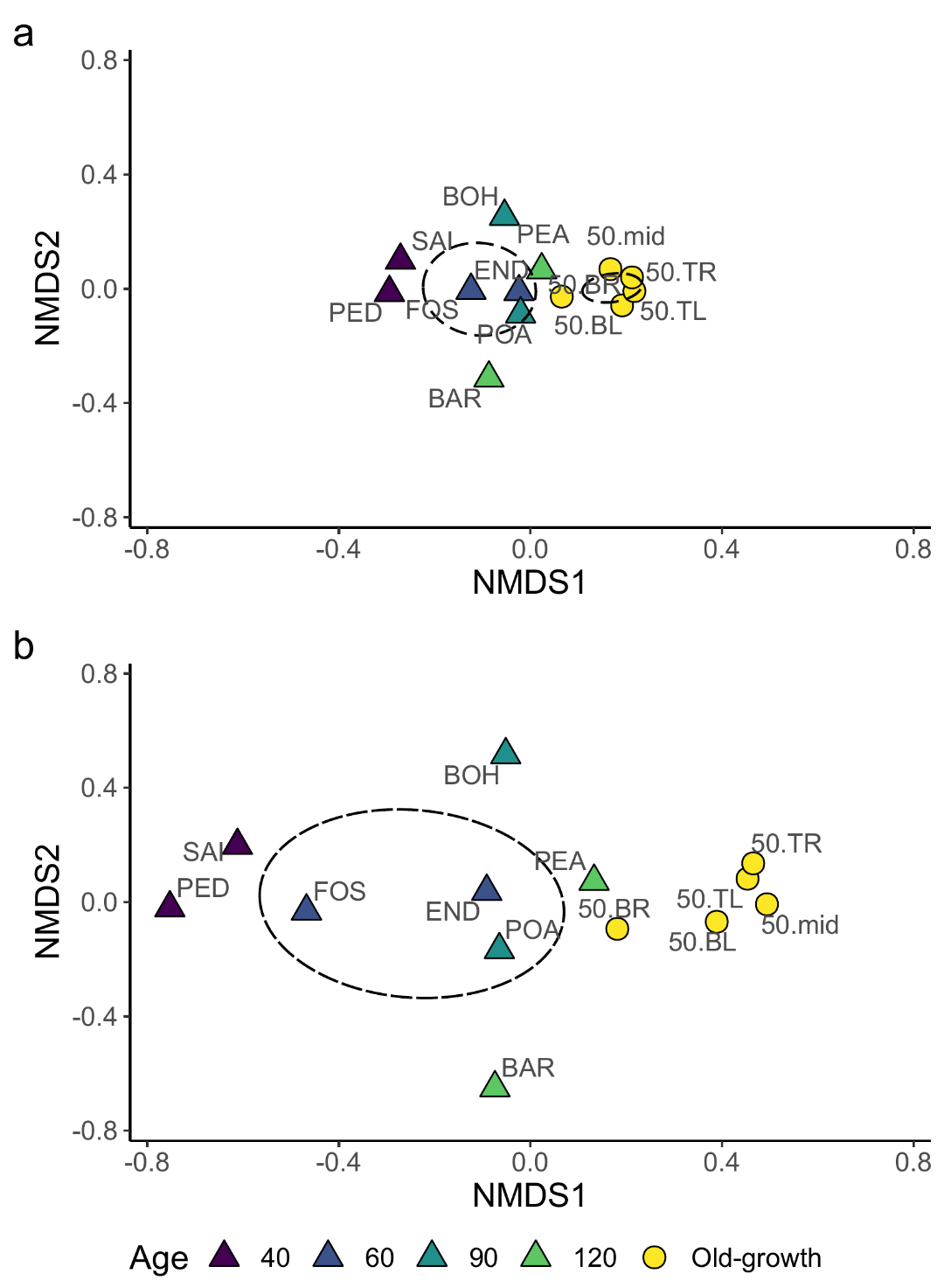


**Figure S5**. Non-metric multidimensional scaling (NMDS) graphs showing the species composition of the secondary and old-growth forest plots in two dimensions for: **a.** NMDS generated using Sorensen similarity index and **b.** NMDS generated using Morisita-Horn similarity index. Points (plots) which are closer together in ordination space are more similar and the dashed lines indicate 95% confidence ellipses for secondary and old-growth forest. Plots are labelled with their three letter abbreviations (Table S1).

**Table S9**. The five most abundant midstory and canopy tree or palm species ≥ 5 cm per 1-ha plot. Midstory or canopy species were identified by their maximum height (Condit et al., 2010; Croat, 1978) and were included to allow direct comparison with Dent et al. (2013).

| Age | Plot | Species | Number of individuals | Percent total individuals (%) |
| --- | --- | --- | --- | --- |
| 40 years | **Pedro Gomez** | *Malouetia guatemalensis* | 58 | 8.68 |
|  |  | *Xylopia frutescens* | 35 | 5.24 |
|  |  | *Protium panamense* | 34 | 5.09 |
|  |  | *Alseis blackiana* | 32 | 4.79 |
|  |  | *Miconia argentea* | 29 | 4.34 |
| 40 years | **Saino** | *Gustavia superba* | 74 | 12.63 |
|  |  | *Miconia argentea* | 56 | 9.56 |
|  |  | *Inga sapindoides* | 55 | 9.39 |
|  |  | *Spondias radlkoferi* | 43 | 7.34 |
|  |  | *Astrocaryum standleyanum* | 26 | 4.44 |
| 60 years | **Enders** | *Oenocarpus mapora* | 116 | 15.03 |
|  |  | *Gustavia superba* | 60 | 7.77 |
|  |  | *Alseis blackiana* | 46 | 5.96 |
|  |  | *Trichilia tuberculata* | 46 | 5.96 |
|  |  | *Xylopia macrantha* | 46 | 5.96 |
| 60 years | **Foster's** | *Inga vera* | 58 | 10.21 |
|  |  | *Alseis blackiana* | 50 | 8.80 |
|  |  | *Myrciaria floribunda* | 43 | 7.57 |
|  |  | *Heisteria concinna* | 40 | 7.04 |
|  |  | *Cordia alliodora* | 36 | 6.34 |
| 90 years | **Bohio** | *Gustavia superba* | 53 | 8.97 |
|  |  | *Virola sebifera* | 47 | 7.95 |
|  |  | *Maquira guianensis* | 45 | 7.61 |
|  |  | *Protium panamense* | 40 | 6.77 |
|  |  | *Brosimium alicastrum* | 38 | 6.43 |
| 90 years | **Poachers** | *Trichilia tuberculata* | 106 | 13.71 |
|  |  | *Protium panamense* | 92 | 11.90 |
|  |  | *Oenocarpus mapora* | 55 | 7.12 |
|  |  | *Xylopia macrantha* | 50 | 6.47 |
|  |  | *Tetragastris panamensis* | 42 | 5.43 |
| 120 years | **Barbour** | *Gustavia superba* | 384 | 47.70 |
|  |  | *Oenocarpus mapora* | 58 | 7.20 |
|  |  | *Alseis blackiana* | 34 | 4.22 |
|  |  | *Luehea seemannii* | 34 | 4.22 |
|  |  | *Protium panamense* | 31 | 3.85 |
| 120 years | **Pearson** | *Trichilia tuberculata* | 63 | 10.77 |
|  |  | *Oenocarpus mapora* | 48 | 8.21 |
|  |  | *Astrocaryum standleyanum* | 43 | 7.35 |
|  |  | *Hirtella triandra* | 42 | 7.18 |
|  |  | *Gustavia superba* | 33 | 5.64 |
| Old-growth | **50-ha BL** | *Alseis blackiana* | 49 | 6.23 |
|  |  | *Oenocarpus mapora* | 46 | 5.85 |
|  |  | *Prioria copaifera* | 27 | 3.44 |
|  |  | *Trichilia tuberculata* | 27 | 3.44 |
|  |  | *Protium tenuifolium* | 25 | 3.18 |
| Old-growth | **50-ha BR** | *Oenocarpus mapora* | 72 | 11.03 |
|  |  | *Drypetes standleyi* | 53 | 8.12 |
|  |  | *Alseis blackiana* | 51 | 7.81 |
|  |  | *Hirtella triandra* | 48 | 7.35 |
|  |  | *Tetragastris panamensis* | 42 | 6.43 |
| Old-growth | **50-ha Mid** | *Trichilia tuberculata* | 48 | 8.87 |
|  |  | *Oenocarpus mapora* | 35 | 6.47 |
|  |  | *Tetragastris panamensis* | 35 | 6.47 |
|  |  | *Quararibea asterolepis* | 26 | 4.81 |
|  |  | *Hirtella triandra* | 20 | 3.70 |
| Old-growth | **50-ha TL** | *Socratea exorrhiza* | 57 | 7.79 |
|  |  | *Alseis blackiana* | 50 | 6.83 |
|  |  | *Oenocarpus mapora* | 40 | 5.46 |
|  |  | *Tetragastris panamensis* | 26 | 3.55 |
|  |  | *Protium panamense* | 20 | 2.73 |
| Old-growth | **50-ha TR** | *Hirtella triandra* | 55 | 6.58 |
|  |  | *Protium tenuifolium* | 55 | 6.58 |
|  |  | *Drypetes standleyi* | 43 | 5.14 |
|  |  | *Oenocarpus mapora* | 39 | 4.67 |
|  |  | *Poulsenia armata* | 38 | 4.55 |


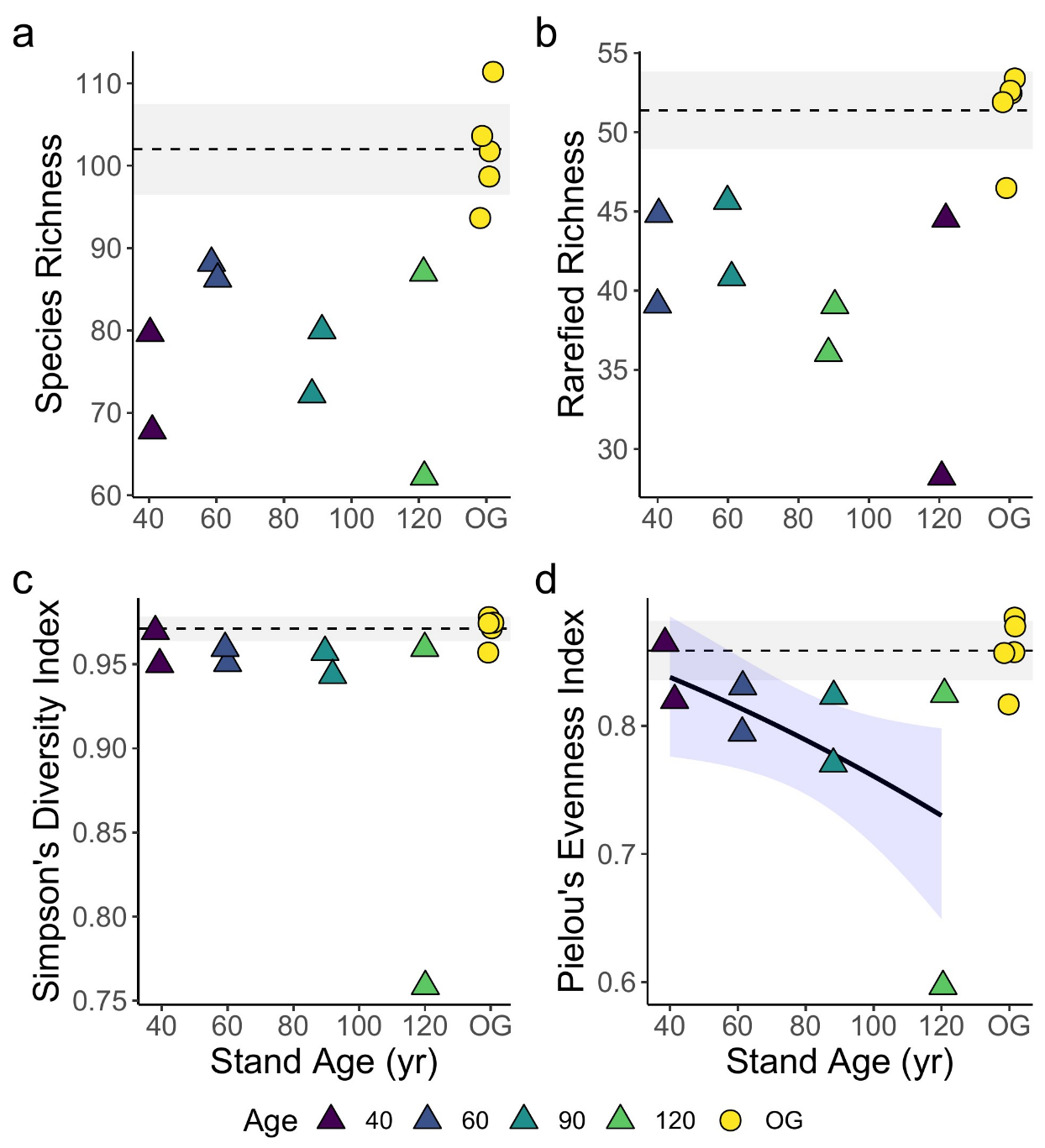


**Figure S6.** Richness and diversity metrics per plot plotted against stand age for all canopy and midstory trees and palms ≥ 5cm. **a.** Species richness; **b.** Rarefied species richness; **c.** Simpson’s diversity index and **d.** Pielou’s evenness index. The mean values of old-growth plots are indicated by the dashed lines; grey shading indicates 95% confidence intervals. Model predictions (+/- 95% C.I.) are plotted in blue shading for models with significant age effects (using the same model selection criteria as described above). These data use the same tree inclusion criteria as Dent et al. (2013), i.e., understory trees and shrubs were excluded prior to analysis. Rarefied richness was also rarefied to 120 stems to match Dent et al. (2013).

**References:**

Baillie, I., Elsenbeer, H., Barthold, F., Grimm, R., & Stallard, R. (2006). *Semi-detailed soil survey of Barro Colorado Island, Panama*. Retrieved from https://striresearch.si.edu/bci-soil-map/

Bartoń, K. (2022). *MuMIn: Multi-model inference*. R package version 1.47.1. Retrieved from https://cran.r-project.org/package=MuMIn

Condit, R., Pérez, R., & Daguerre, N. (2010). *Trees of Panama and Costa Rica*. Princeton University Press.

Cribari-Neto, F., & Zeileis, A. (2010). Beta Regression in R. *Journal of Statistical Software*, *34*(2), 1–24. https://doi.org/10.18637/jss.v069.i12

Croat, T. B. (1978). *Flora of Barro Colorado Island*. Stanford, California: Stanford University Press. https://doi.org/10.2307/1219834

Denslow, J. S., & Guzman, S. G. (2000). Variation in stand structure, light and seedling abundance across a tropical moist forest chronosequence, Panama. *Journal of Vegetation Science*, *11*(2), 201–212. https://doi.org/10.2307/3236800

Dent, D. H., DeWalt, S. J., & Denslow, J. S. (2013). Secondary forests of central Panama increase in similarity to old-growth forest over time in shade tolerance but not species composition. *Journal of Vegetation Science*, *24*(3), 530–542. https://doi.org/10.1111/j.1654-1103.2012.01482.x

Dent, D. H., & Elsy, A. D. (*In press*). Structure, diversity and composition of secondary forests of the Barro Colorado Nature Monument. In H. C. Muller-Landau & S. J. Wright (Eds.), *The First 100 Years of Research on Barro Colorado: Plant and Ecosystem*. Smithsonian Institution Scholarly Press.

Enders, R. K. (1935). Mammalian life histories from Barro Colorado Island, Panama. *Bulletin of the Museum of Comparative Zoology at Harvard College*, *78*(4), 385–502.

Gelman, A., & Su, Y.-S. (2022). *arm: Data analysis using regression and multilevel/hierarchical models*. R package version 1.13-1. Retrieved from https://cran.r-project.org/package=arm

Hartig, F. (2022). *DHARMa: Residual diagnostics for hierarchical (multi-level/mixed) regression models*. R package version 0.4.6. Retrieved from https://cran.r-project.org/package=DHARMa

Hijmans, R., Kapoor, J., Wieczorek, J., Garcia, N., Maunahan, A., Rala, A., & Mandel, A. (2020). Global Administrative Areas. Retrieved April 29, 2020, from https://gadm.org/download_country.html

Jones, I. L., DeWalt, S. J., Lopez, O. R., Bunnefeld, L., Pattison, Z., & Dent, D. H. (2019). Above- and belowground carbon stocks are decoupled in secondary tropical forests and are positively related to forest age and soil nutrients respectively. *Science of The Total Environment*, *697*, 133987. https://doi.org/10.1016/j.scitotenv.2019.133987

Kenoyer, L. A. (1929). General and successional ecology of the lower tropical rain-forest at Barro Colorado Island, Panama. *Ecology*, *10*(2), 201–222.

Lüdecke, D., Ben-Shachar, M. S., Patil, I., Waggoner, P., & Makowski, D. (2021). performance: An R package for assessment, comparison and testing of statistical models. *Journal of Open Source Software*, *6*(60), 3139. https://doi.org/10.21105/joss.03139

Oksanen, J., Blanchet, F. G., Friendly, M., Kindt, R., Legendre, P., McGlinn, D., … Wagner, H. (2022). vegan: Community ecology package. R package version 2.6-4. Retrieved from https://cran.r-project.org/package=vegan

QGIS Development Team. (2020). *QGIS Geographic Information System*. Open Source Geospatial Foundation Project. Retrieved from http://qgis.osgeo.org

Schielzeth, H. (2010). Simple means to improve the interpretability of regression coefficients. *Methods in Ecology and Evolution*, *1*(2), 103–113. https://doi.org/10.1111/j.2041-210x.2010.00012.x

Smithsonian Tropical Research Institute (STRI). (2019). STRI GIS Data Portal. Retrieved June 10, 2019, from https://stridata-si.opendata.arcgis.com/

Smithsonian Tropical Research Institute (STRI). (2020). Barro Colorado Natural Monument DTM - LiDAR. Retrieved June 26, 2020, from https://stridata-si.opendata.arcgis.com/datasets/barro-colorado-natural-monument-dtm-lidar-

Wolf, J. A., Hubbell, S. P., Fricker, G. A., & Turner, B. L. (2015). Geospatial observations on tropical forest surface soil chemistry. *Ecology*, *96*(8), 2313–2313. https://doi.org/10.1890/15-0558.1
